# Giant infrared bulk photovoltaic effect in tellurene for broad-spectrum neuromodulation

**DOI:** 10.1038/s41377-024-01640-w

**Published:** 2024-09-27

**Authors:** Zhen Wang, Chunhua Tan, Meng Peng, Yiye Yu, Fang Zhong, Peng Wang, Ting He, Yang Wang, Zhenhan Zhang, Runzhang Xie, Fang Wang, Shuijin He, Peng Zhou, Weida Hu

**Affiliations:** 1 State Key Laboratory of Infrared Physics, Shanghai Institute of Technical Physics, Chinese Academy of Sciences, Shanghai, China; 2 State Key Laboratory of ASIC and System, Department of Microelectronics, Fudan University, Shanghai, China; 3 University of Chinese Academy of Sciences, Beijing, China; 4 School of Life Science and Technology, ShanghaiTech University, Shanghai, China; 5 School of Mathematical and Physical Sciences, Wuhan Textile University, Wuhan, China; 6 Shanghai Clinical Research and Trial Center, Shanghai, China

**Keywords:** Optical sensors, Optoelectronic devices and components

## Abstract

Given the surpassing of the Shockley-Quiesser efficiency limit in conventional p-n junction photovoltaic effect, bulk photovoltaic effect (BPVE) has garnered significant research interest. However, the BPVE primarily focuses on a narrow wavelength range, limiting its potential applications. Here we report a giant infrared bulk photovoltaic effect in tellurene (Te) for broad-spectrum neuromodulation. The generated photocurrent in uniformly illuminated Te excludes other photoelectric effects and is attributed to the BPVE. The bulk photovoltaic wavelength in Te spans a wide range from the ultraviolet (390 nm) to the mid-infrared (3.8 µm). Moreover, the photocurrent density of 70.4 A cm^−2^ under infrared light simulation outperforms that in previous ultraviolet and visible semiconductors as well as infrared semimetals. Te attached to the dendrites or somata of the cortical neurons successfully elicit action potentials under broad-spectrum light irradiation. This work lays the foundation for the further development of infrared BPVE in narrow bandgap materials.

## Abstract (web summary)

Giant infrared bulk photovoltaic effect is observed in tellurene and applied in neuromodulation.

## Graphical abstract

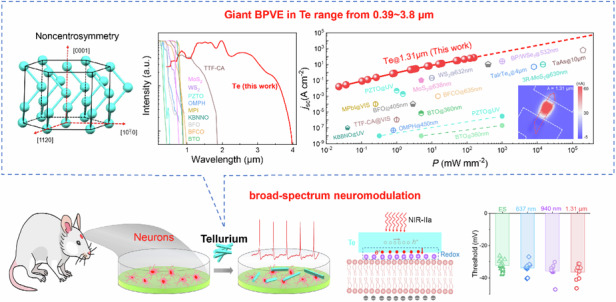

## Introduction

Efficient light-to-electricity conversion is pivotal in diverse applications such as imaging, free-space communication, biological sensing, and clean energy^1,2^. The BPVE, a second-order optical effect, has emerged as a subject of intense exploration due to its potential to overcome the Shockley-Quiesser efficiency limit inherent in the traditional p-n junction photovoltaic effect^3^. The initial observation of BPVE in ferroelectric oxide materials (LiNbO_3_, BaTiO_3_, and Pb(Zr_x_Ti_1-x_)O_3_) offers unprecedented opportunities for extensive investigations across various materials^4–6^. Beyond ferroelectric oxide materials, recent studies have delved into ferroelectric superlattices^7^, perovskite-type halides^8^, organic crystals^9^, semimetals^10,11^, and van der Waals materials^12–14^. Notably, van der Waals materials with low dimensionality, strong symmetry breaking, and high strain compatibility have demonstrated exceptional BPVE characteristics^15^. For instance, the in-plane tensile strain disrupts the inversion symmetry of rhombohedral-type MoS_2_, leading to a remarkable short-circuit photocurrent of 10 A cm^−2^ under 630 nm light^12^. Due to the interband optical transitions in semiconductors and heterostructures, the current BPVE response mainly focuses on a limited wavelength range from ultraviolet to visible. Although Berry curvature and scattering in semimetals can assist infrared BPVE generation, the optoelectronic transition only produced from the polarized single-wavelength laser light is in a low probability and severely hampers further applications^10^. The exploration of broadband BPVE remains a significant challenge.

The photoelectric effect used in optical neuromodulation presents a compelling avenue for achieving minimally invasive and remotely controlled stimulation of neurons^16,17^. This strategy encompasses four distinct modalities: p-n junction photovoltaic effect, spectrum-selective upconversion, photothermal effect, and photoacoustic effect. Neuromodulation through the p-n junction photovoltaic effect, employing coaxial silicon nanowires or organic semiconductors, is characterized by minimal heat generation^18–20^. Visible light (532 nm) is utilized to achieve the modulation of neurons. Nanoparticles with spectrum-selective upconversion demonstrate the capability to convert near-infrared (NIR) light to visible light^21^. Presently, the simulation wavelength falls within the range of 800 nm to 980 nm. Furthermore, NIR light can induce a photothermal effect, elevating the temperature of materials such as poly(benzobisthiadiazole-alt-vinylene) (pBBTV) nanoparticles, gold nanorods, and mesostructured silicon^22–24^. Under 1.06 µm light illumination, pBBTV nanoparticles could elicit action potentials in neurons. The photoacoustic effect, observed in nanoparticles, involves 1.03 µm light irradiation during optical neuromodulation^25,26^. As a result, the current simulation for optical neuromodulation is also confined to a narrow wavelength range. Exploring the realization of broad-spectrum neuromodulation through alternative photoelectric effects remains a valuable and intriguing avenue.

Here, we discover a giant infrared BPVE in Te. Te with controllable lengths ranging from 0.95 to 12.92 μm exhibit significant advantages in both bulk photovoltaic wavelength and photocurrent density, outperforming previous semiconductors and semimetals. Leveraging the exceptional infrared BPVE observed in our experiments, we successfully achieve broad-spectrum neuromodulation spanning from the visible (637 nm) to the infrared (940 nm, 1.31 µm, and 1.55 µm) using Te nanoflakes. Our findings indicate that the infrared BPVE in narrow bandgap nanomaterials not only enhances the efficiency of converting broadband light to electric power but also provides a novel strategy for remotely stimulating therapeutics.

## Results

### Growth and characterization of Te

**Fig. 1:**
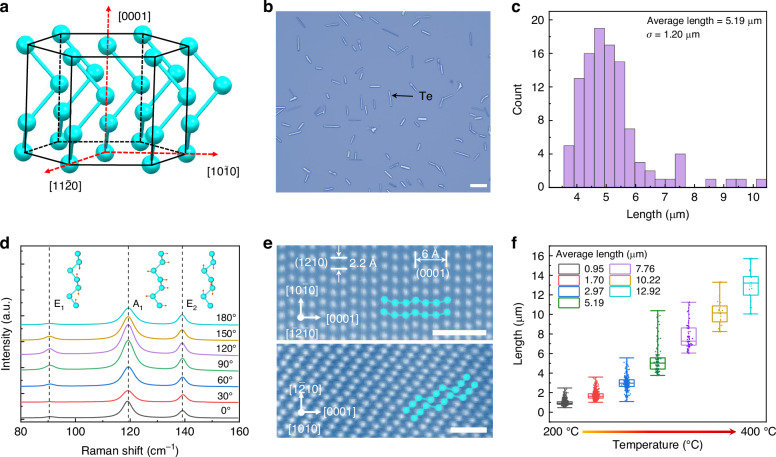
Growth and characterization of Te. **a** Atomic structure schematic of Te. **b**, **c** Optical microscope image and length distribution analysis of synthesized Te. The growth region temperature on the substrate is ~300 °C. The lengths are extracted and measured from corresponding optical images. σ is the standard deviation of the length, respectively. Scale bar represents 10 µm. **d** Polarized Raman spectra of Te. Raman active modes are discernible at the peaks of 90.4 cm^−1^, 119.2 cm^−1^, and 139.6 cm^−1^, denoted as E_1_, A_1_, and E_2_. A_1_ mode results from chain expansion, where each atom undergoes vibrational motion within the basal plane. In contrast, the E_1_ and E_2_ modes correspond to bond-bending and asymmetric stretching. **e** Atomically resolved STEM images of Te obtained from different crystal planes. The width of Te is bigger than 1 µm. Cyan balls represent Te atoms. White scale bars represent 1 nm. **f** Length distribution of Te as the relationship of the growth region temperature

Te is an air-stable narrow bandgap semiconductor and possesses the ability to absorb infrared light^27–29^. Each Te atom is covalently bonded with two nearest neighbors and forms a helical chain. A Te chain is surrounded by and stacked with six other Te chains and bonded into a hexagonal structure by weak van der Waals forces (Fig. 1a). Te belongs to the *P*21 space group with a noncentrosymmetry^30^. In this work, Te was synthesized on SiO_2_/Si substrates using chemical vapor deposition (Supplementary Fig. 1). The SnTe_2_ source was heated to 600 °C and maintained at that temperature for 30 min. By controlling the growth region temperature on the substrate to approximately 300 °C, Te with an average length of 5.19 µm were successfully obtained (Fig. 1b, c). The synthesized materials were characterized using polarized Raman microscopy, revealing three distinct peaks at 90.4 cm^−1^, 119.2 cm^−1^, and 139.6 cm^−1^, which correspond to the Raman active modes (E_1_, A_1_, and E_2_) of Te (Fig. 1d). The observed changes in the *E*_1_ mode intensity at angles of 0°, 30°, and 180° can be attributed to the anisotropic properties of Te, particularly its chiral-chain van der Waals structure^31^. This unique structure leads to variations in lattice deformation and intra-chain atomic displacement with changing angles, which in turn affects the Raman mode intensities. Our synthesized Te nanoflakes possess a good crystalline quality and an absorption cutoff wavelength of 3.8 µm (Supplementary Fig. 2). Meanwhile, Te nanoflakes exhibited high surface quality and featured chains connected by van der Waals bonds (Fig. 1d and Supplementary Figs. 3 and 4). The interplanar spacings of 6.0 Å and 2.2 Å correspond to those of the (0001) and ($1\bar{2}10$) planes, respectively. The growth process of Te was found to be dependent on the relationship between the atomic migration rate and temperature (Supplementary Note 1). As the temperature increased, the atomic migration rate exponentially increased as well. This temperature-induced rise in thermal energy enabled the atoms or ions to overcome activation energy or potential barriers, thereby accelerating the growth rate of Te. By regulating the temperature of the growth region on the substrate from 200 °C to 400 °C, we observed a corresponding change in the length of Te from 0.95 μm to 12.92 μm (Fig. 1f and Supplementary Fig. 5).

### Infrared photoelectric response of Te

**Fig. 2:**
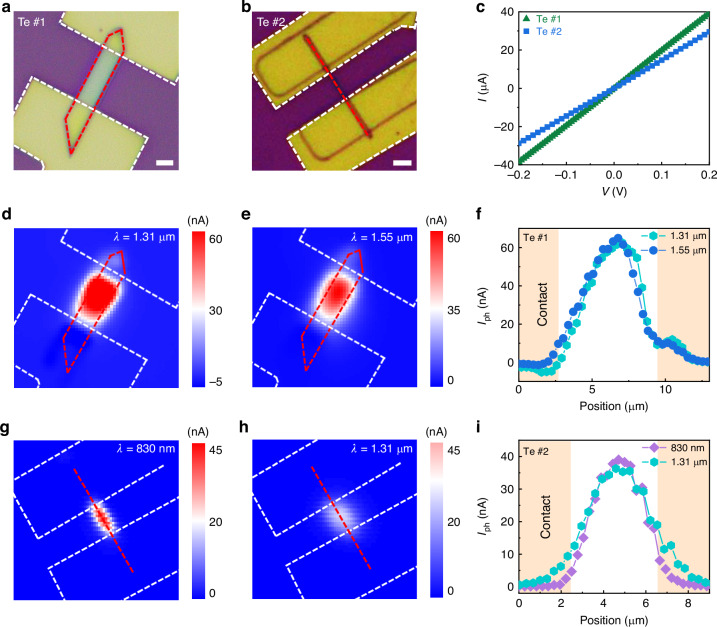
Infrared photoelectric response of Te. **a**, **b** Optical microscope images of Te devices #1 and #2. Boundaries of Te and contact electrodes are outlined by dashed red and white lines, respectively. The width of Te devices #1 and #2 are great and less than 1 µm, respectively. Scale bars represent 5 µm. **c** *I*-*V* curves of Te devices #1 and #2, demonstrating that good contact between Te and platinum/gold formed. **d**, **e** SPM of Te device #1 under 1.31 µm and 1.55 µm laser illumination. Power densities are 1.64 and 0.65 mW mm^−2^, respectively. Shapes or boundaries of the Te and the corresponding contact electrodes are marked by the dashed red and white line regions, respectively. **f** *I*_ph_ profile extracted along the center line of the Te channel in **d** and **e**. **g**, **h** SPM of Te device #2 under 830 nm and 1.31 µm laser illumination. Power densities are 0.12 and 0.041 mW mm^−2^, respectively. The dashed red and white lines represent the Te and the contact electrode areas, respectively. **i** *I*_ph_ profile extracted along the center lines of Te channel in **g** and **h**. SPM of Te devices was measured at zero voltage. All measurements of Te devices in this work were carried out at room temperature and in an air atmosphere unless otherwise stated

We successfully fabricated Te devices with different sizes (Supplementary Note 2). Optical microscope images of the fabricated Te devices with different widths are presented in Fig. 2a, b. After conducting electrical measurements, the linear *I*-*V* curves of Te devices indicate that good contact between Te and platinum/gold is achieved (Fig. 2c).

Here, scanning photocurrent mapping (SPM) is used to characterize the photoelectric response of Te devices at an applied voltage of 0 V. The SPM characterization results of Te device #1, with a width greater than 1 µm, under 637 nm, 830 nm, 1.31 µm, 1.55 µm, and 2 µm laser illumination are shown in Fig. 2d, e, and Supplementary Fig. 6a–c, respectively. Notably, an observed photocurrent from the visible to the infrared is generated in the Te channel. Photocurrent (*I*_ph_) profiles extracted from the corresponding SPM images further show that a larger photocurrent appears in the Te channel rather than in the metal contact interface regions (Fig. 2f and Supplementary Fig. 6d,f). The *I*_ph_ away from one contact electrode gradually increases and almost reaches a maximum in the middle of the Te channel. The photocurrent generated at the contact interface regions exhibited an opposite behavior to that of the Te channel when the incident light varied from the visible to the shortwave infrared. Negative *I*_ph_ values are observed in the contact interface regions under 637 nm, 830 nm, and 1.31 µm light illumination, while *I*_ph_ becomes positive under 1.55 µm and 2 µm light illumination. However, importantly, the *I*_ph_ generated in the Te channel is consistently much larger than the negative *I*_ph_ in the interfaces, indicating that the observed photoelectric response in the Te channel is dominant for both the visible and infrared regions.

The SPM and *I*_ph_ profiles of Te device #2, with a width less than 1 µm, are displayed in Fig. 2g, h and Supplementary Fig. 7. Similar to Te device #1, the *I*_ph_ tends to be produced in the Te channel. In contrast to Te device #1, no negative *I*_ph_ is observed in the contact region of device #2 under both visible and infrared light illumination. Overall, a room-temperature and intrinsic photoelectric response is demonstrated in homogeneous single-component Te.

**Fig. 3:**
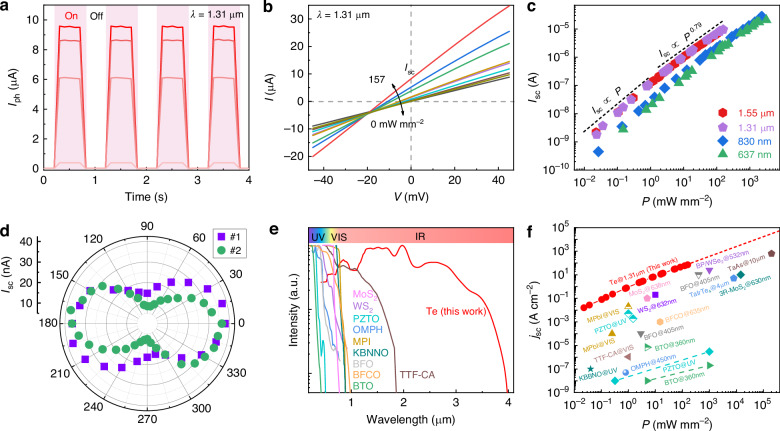
Bulk photovoltaic response in Te devices and overview of the BPVE in reported materials. **a** Time-resolved *I*_ph_ of Te device #1 under 1.3 µm laser illumination with different power densities. The power densities are 157, 126, 83.9, and 3.5 mW mm^−2^. The applied voltage is zero. **b** *I*-*V* curves of Te device #1 under 1.3 µm laser illumination with the power density changing from 0 to 157 mW mm^−2^. **c** *I*_sc_ versus light power in Te device #1 for visible and infrared wavelengths. **d** Infrared linear polarization dependence of *I*_sc_ in Te devices #1 and #2. The light source wavelength is 1.31 µm. **e** Photoresponse spectra of various semiconductors with the BPVE. The data of this work is based on the measurement result of Te device #1. References are shown in Supplementary Information Table 3. **f** Power dependence of *j*_*sc*_ in reported materials for different light wavelengths. The data of this work is based on the measurement result of Te device #1. References can be found in Supplementary Information Table 3

The time-resolved *I*_ph_ of the Te devices obtained an applied voltage of 0 V are shown in Fig. 3a and Supplementary Figs. 8a–c and 9a–c. Upon activation of the visible and infrared light sources, a steady *I*_ph_ is rapidly generated. Moreover, the generated *I*_ph_ reaches up to several tens of microamperes. Under dark conditions, the *I*-*V* curves of the Te devices pass through the origin point (Fig. 3b and Supplementary Fig. 8d). As the power density of the incident light becomes large, both the short-circuit current (*I*_sc_) and open-circuit voltage simultaneously increase and shift away from the origin point. This nonzero characteristic of the Te devices under light illumination is likely attributed to the photovoltaic effect. However, such a linear characteristic of Te devices fairly differs from the exponential curve observed in p-n junction devices^32^.

### Bulk photovoltaic effect of Te

Figure 3c and Supplementary Fig. 9d display the light power dependence of *I*_sc_ in Te devices #1 and #2 at four different wavelengths. For the same power, *I*_sc_ for the shortwave infrared region is larger than that for the visible region, which is attributed to the variation in the absorption coefficient of Te at different wavelengths^33,34^. Interestingly, as the light power increases, the power dependence of the Te devices transitions from linear to sublinear. This transition cannot be explained by the Schottky barrier photovoltaic effect, which typically exhibits a larger linear range in the contact electrode regions^35^.

Photoelectric effects are fundamental in optoelectronics. We summarize various photoelectric effects and differentiate them to identify the photoelectric response mechanism in Te devices (Supplementary Table 1 and Supplementary Note 3). P-n junction and Schottky barrier photovoltaic effects are excluded from the aspects of the linear dark *I*-*V* curve, power-dependent *I*_sc_, device structure. Since the Te devices operate under an applied voltage of 0 V, the photoconductive effect is not responsible for the observed photocurrent. The photothermoelectric effect would result in an opposite photocurrent, which contradicts the single response observed in the Te channels. While the flexo-photovoltaic effect exhibits a linear dark *I*-*V* curve and a nonzero photocurrent in the channel, it requires external strain in the measuring devices. The photo-Dember effect occurs on inhomogeneous sample surfaces^13,36^. The contribution of photogalvanic and photon drag effect is very small for the photovoltaic response in the Te (Supplementary Note 4 and Supplementary Fig. 10). The results of Te don’t accord with these mechanisms. Thus, the observed photoelectric effect in Te is the BPVE. Photocurrent change profiles of Te device #1 are attributed to different photoelectric effects under different wavelengths of light (Supplementary Fig. 11). Under light illumination, a negative current at one electrode and a positive current at the other electrode of Te are observed and caused by the photothermoelectric effect^37^. Positive photocurrent of Te channel is extracted and is due to intrinsic BPVE. The absorption coefficient of Te changes with different wavelengths^33,34^. The BPVE-based photocurrent in Te for the shortwave infrared region (1.55 µm and 2 µm) is larger than that for the other regions (637 nm, 830 nm, and 1.31 µm). For 637 nm, 830 nm, and 1.31 µm light, negative current produced by the photothermoelectric effect surpasses the current induced by the BPVE. As a result, the area at one electrode demonstrates a negative photocurrent response. Conversely, under 1.55 µm and 2 µm light illumination, the BPVE predominates, yielding an overall positive current response.

Light polarization dependence of *I*_ph_ is another critical feature of the BPVE, which is determined by the nature of noncentrosymmetric crystals^30,38^. This characteristic of the Te devices is clearly shown in a polar diagram (Fig. 3d). As the linear polarization degree changes, the *I*_ph_ in the Te devices exhibits a strong anisotropic behavior. To further confirm the BPVE in the Te, other batches of Te devices with varying widths are fabricated and characterized, indicating that the BPVE is well reproduced (Supplementary Figs. 12–17). Photocurrent increases induced by the BPVE with the increasing photosensitive area (the product of the length and width) of the Te nanoflake device (Supplementary Table 2). These results demonstrate that the observed photoresponse in Te is attributable to the BPVE. This BPVE in Te nanoflakes is primarily attributed to the asymmetric properties^30^. Notably, the BPVE in the Te devices is quite outstanding quantitively (Supplementary Note 5 and Supplementary Information Table 3). The bulk photovoltaic response of Te spans a wide range, from the ultraviolet (390 nm) to the mid-infrared (3.8 µm), a significant enhancement compared to the ultraviolet and visible ranges observed in previous semiconductors (Fig. 3e). Furthermore, the photocurrent density in Te under 1.31 µm light illumination attains an impressive 70.4 A cm^−2^. This elevated photocurrent density in the infrared range not only competes favorably with state-of-the-art materials designed for ultraviolet and visible light but also surpasses that of infrared semimetals (Fig. 3f).

### Broad-spectrum neuromodulation based on Te

**Fig. 4:**
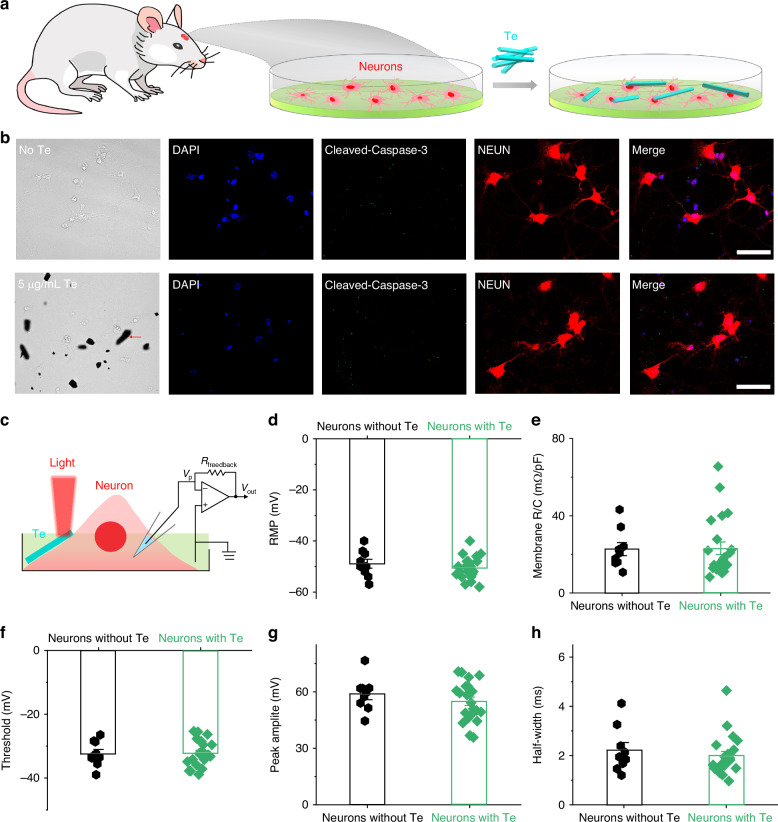
Te does not affect neuronal membrane properties. **a** Co-culture process of mouse primary cortical neurons and Te nanoflakes. Red and cyan shapes represent mouse primary cortical neurons and Te nanoflakes, respectively. **b** No apoptosis observed for neurons co-cultured with Te nanoflakes. **c** Whole-cell patch-clamp recording diagram of a DIV11 primary cortical neuron under broadband light illumination. **d**–**h** No difference in resting membrane potential (RMP), membrane resistance, action potential threshold, amplitude or half-width between primary cortical neurons cultured without and with Te nanoflakes at DIV11. Action potentials were evoked by currents injection through recording pipettes. Membrane R/C was calculated by normalizing membrane resistance to capacitance. Replicate n number represents the number of neurons. Data are presented as mean ± SEM

Te exhibits an intriguing BPVE and directly converts light to electricity. This characteristic of no external voltage is essential for broad-spectrum neuromodulation. To test whether broadband light could directly modulate the activity of a neuron through Te nanoflakes, the primary cortex neurons were extracted from the embryonic day 18 mouse cortex and the Te nanoflakes were added into the culture medium on days in vitro (DIV) 10 (Fig. 4a). The concentration of Te nanoflakes is less than 5 µg/mL, referring to the concentration used in the previous work^39,40^. At DIV 9, we observed a neuron-glia culture composed of neurons, oligodendrocytes (OLIG2^+^ cells), astrocytes (S100*β*^+^) and possibly microglial cells (NeuN^−^ OLIG2^−^ or NeuN^−^ S100*β*^−^), promoting neural maturation in an in vitro culture (Supplementary Fig. 18). After 24 h co-incubation of mouse primary cortical neurons and Te nanoflakes, we observed Te nanoflakes attached to the dendrites or somata of the cortical neurons (Fig. 4b and Supplementary Figs. 19–21). Immunocytochemistry revealed no expression of cleaved Caspase-3 (CAPS-3), an apoptotic marker, in neurons 48 h after co-incubation with Te nanoflakes (Fig. 4b), suggesting no apoptosis induced by Te nanoflakes. Moreover, Te nanoflakes do not degrade in the ACSF solution even after 21 days (Supplementary Note 6 and Supplementary Figs. 22 and 23). We then performed whole-cell patch clamping recordings of the cultured cortical neurons at DIV11 (Fig. 4c). Initially, we found no difference in membrane resistance, resting membrane potential (RMP), action potential (AP) threshold, AP amplitude, or AP half width between neurons cultured with Te and neurons cultured without Te (Supplementary Fig. 24), suggesting that Te do not affect cell membrane properties or neuronal maturation during 48 h culture (Fig. 4d–h). Additionally, no significant changes in pH of ACSF solution, cleaved caspase-3 staining, or the RMP of neurons were observed in neurons after long illumination cycles (Supplementary Note 7 and Supplementary Figs. 25–27)

**Fig. 5:**
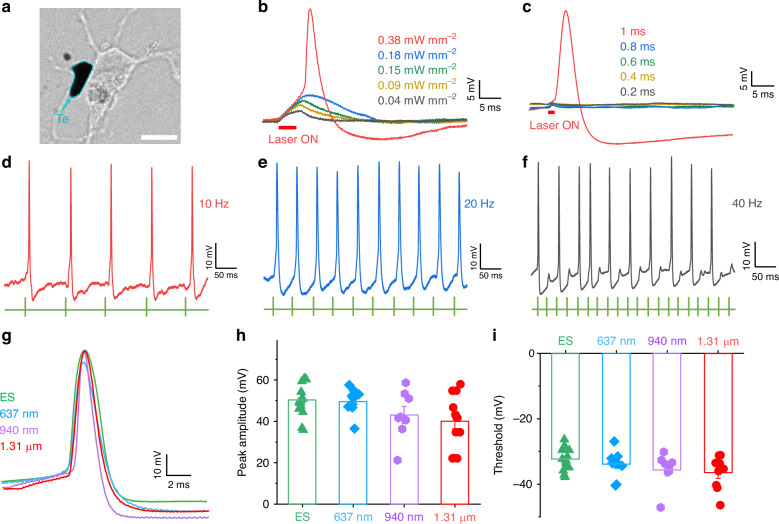
Broad-spectrum regulation of mouse primary cortical neurons based on Te. **a** Microscopy image of primary cortical neuron and Te nanoflake under broadband light illumination. Scale bar represents 10 µm. **b** Electrophysiological current-clamp recording traces of changes in the membrane voltage in the primary cortical neuron co-cultured with Te nanoflake stimulated by 1.31 µm light with different energy densities. The frequency and duration are 10 Hz and 5 ms, respectively. **c** Electrophysiological current-clamp recording traces of changes in the membrane voltage in the primary cortical neuron co-cultured with Te nanoflake stimulated by 1.31 µm light with different durations. The frequency and energy density are 10 Hz and 0.38 mW mm^−2^, respectively. **d**–**f** Electrophysiological recording traces of changes in the membrane voltage in the primary cortical neurons co-cultured with Te nanoflake stimulated by 1.31 µm light with different frequencies. The duration and energy density are 5 ms and 0.38 mW mm^−2^, respectively. **g** Sample traces of single action potentials of the primary cortical neurons cocultured with Te nanoflakes stimulated by the light with different wavelengths (637 nm, 940 nm, and 1.31 µm). **h**, **i** Quantification of the peak amplitude and threshold of action potentials generated by primary cortical neuron cocultured with Te nanoflake. Replicate n number represents the number of neurons. Data are presented as mean ± SEM

We next examined neuronal firing by illuminating a single Te nanoflake attached to a cultured neuron with following different wavelength light (637 nm, 940 nm, and 1.31 µm). The return electrode of the Te nanoflake is the ACSF solution and a grounding wire (Supplementary Fig. 28). Spot diameters of 637 nm, 940 nm, and 1.31 µm light are 19.8 µm, 45.2 µm, and 74.6 µm, respectively (Supplementary Fig. 29 and Supplementary Note 8). Cortical neurons cultured without Te served as a control. We firstly opted for an optimal protocol including the energy density and the light duration and found that an action potential was reliably evoked in a cultured cortical neuron with Te nanoflake attachment by light illumination of 1.31 µm wavelength light with the duration of 1 ms and the density of 0.38 mW mm^−2^ (Fig. 5a–c). Importantly, a series of action potentials were generated with high temporal fidelity following 10 Hz or 20 Hz light illumination, but spikes were frequently generated following 40 Hz light illumination (Fig. 5d–f). By contrast, none of the action potentials was elicited in neurons without Te by illumination of any of the three wavelength lights (Supplementary Figs. 30–32). We also found that the power density for 940 nm and 1.31 µm light to evoke similar frequency of action potentials was less than that for 637 nm light (Supplementary Figs. 33 and 34), suggesting that the Te nanoflakes exhibit more efficient photoelectric conversion for infrared light. It is interesting to point out that 1.55 µm light was able to evoke action potentials in neurons with Te nanoflakes attachment (Supplementary Fig. 35). In a word, broad-spectrum neuromodulation is realized through Te with giant infrared BPVE.

We further examined the characteristics of light-evoked action potentials and compared them with those of current-evoked action potentials. We found that light-evoked and current-evoked action potentials possessed three classic phases including rapid depolarization, repolarization, and fast after hyperpolarization potential (Fig. 5g). Action potentials evoked by 637 nm, 940 nm, or 1.31 µm light illumination were not different in the threshold and amplitude (Fig. 5h, i). Likewise, the threshold of light-evoked action potentials was comparable to that of current-evoked action potentials. Meanwhile, optical neuromodulation based on Te exhibits good reproducibility from the visible to the infrared, even during a long duration (Supplementary Figs. 36–38). These results together suggest that broad-spectrum neuromodulation can be reliably achieved through Te.

### Broad-spectrum neuromodulation mechanism via the BPVE

After demonstrating the regulation of broadband light using Te nanoflakes, we further investigated the mechanism of neuromodulation. One found that Te adhered to the neuron well when the length of Te ranged from 1 to 3 μm (Supplementary Fig. 39a). However, as the length of Te nanoflakes increased from 4–6 μm to 7–9 μm, the probability of Te nanoflakes moving away from neurons increased from 22% to 64%. Te nanoflakes with a length of 10-15 μm hardly adhered to the neurons after the culture dish was shaken. On the other hand, we found that the modulation probability of neurons under broadband light irradiation depended on the distance between the Te and the soma of the neurons (Supplementary Fig. 39b). When the distance was less than 4 µm, more than 70 percent of the co-cultured neurons with Te nanoflakes could generate action potentials by light illumination. However, as the distance increased to 10–14 µm, the optical modulation probability decreased to 9.5%.

**Fig. 6:**
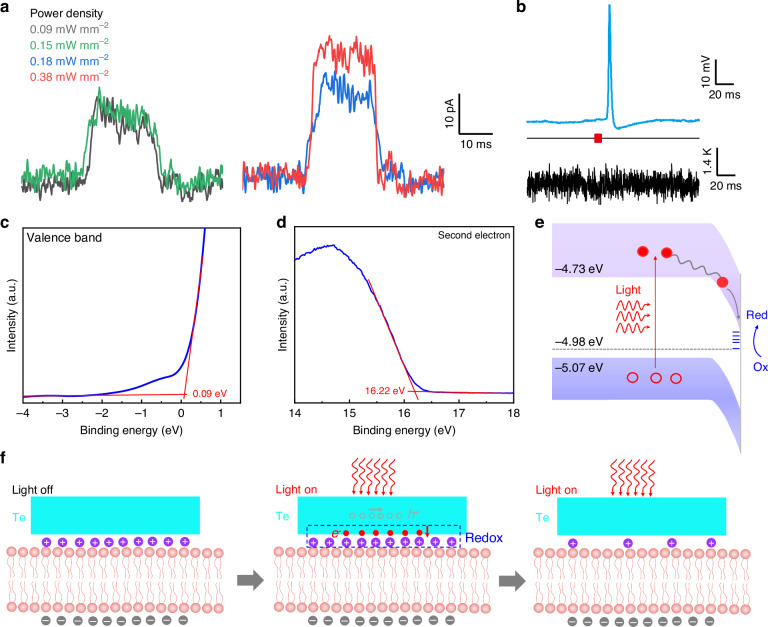
Broad-spectrum neuromodulation mechanism of neuron and Te. **a** Photocurrent traces from a Te nanoflake with 1.31 µm light. The frequency and duration of the light are 1 Hz and 10 ms, respectively. **b** Representative electrophysiological current-clamp recording traces and temperature changes stimulated by 940 nm light. Traces from the recording pipette (top) and the thermometer pipette (bottom). Power densities of 940 nm light are approximately 0.57 mW mm^−2^. **c**, **d** Valence band spectrum and second electron cutoffs of Te nanoflakes. According to W = *hν*−*E*_cut_, where *hν* = 21.2 eV is the photon energy of He I light source, the work function of Te was estimated to be 4.98 eV. The Fermi levels are located 0.09 eV above the valence bands. **e** Band diagram of photoelectric regulation mechanism based on the BPVE of Te nanoflakes. *E*_C_ and *E*_V_ are the energies of the conduction band minimum and valence band maximum, respectively. Red arrows represent the broadband light stimulus. Red circles and rings represent photogenerated electrons and holes, respectively. **f** Broad-spectrum neuromodulation mechanism illustration of neurons and Te. The redox reaction appears at the interface between Te and neurons. Purple and gray balls represent extracellular cations and intracellular anions respectively. Red and gray hollow balls represent the photogenerated electrons and holes, respectively

We employed a patch-clamp configuration to assess the photocurrents of a Te nanoflake responding to light of 637 nm, 940 nm, and 1.31 μm wavelengths in neuromodulation, each lasting 10 ms (Fig. 6a and Supplementary Fig. 40). The Te nanoflake, situated at the tip of a recording pipette, was directly illuminated by the light, and the resulting currents were recorded in the voltage-clamp mode at a holding potential of zero millivolts. The photocurrents induced by light illumination were sustained and non-capacitive, with their amplitudes being directly proportional to the power of the incident light and capable of exceeding 20 pA (Supplementary Note 9). The direction of the currents generated by the Te was consistent with that observed in p-i-n silicon nanowires^18^, indicating a photoelectric reaction occurring on the surface of the Te nanoflake. As the number of cyclic pulses increases up to 1000, the photocurrents generated in the Te nanoflakes rapidly decrease. Beyond 1000 pulses, the photocurrents reach a plateau and no further changes are observed up to 2×10^5^ pulses (Supplementary Fig. 41). No photovoltage was observed in the Te nanoflakes (Supplementary Fig. 42). Furthermore, Te nanoflakes on the SiO_2_/Si substrate were subjected to 60 s illumination with various wavelengths of light (Supplementary Fig. 43 and Supplementary Note 10). Upon comparison, we only observed a maximum temperature change of 0.18 °C. We also conducted a simultaneous measurement of temperature and neuronal action potential produced by laser stimulation (Supplementary Figs. 44 and 45). 940 nm light illumination reliably evokes the action potentials in the neurons, but causes no significant change in the temperature (Fig. 6b), suggesting that the BPVE of Te predominates in broad-spectrum neuromodulation, rather than the photothermal effect.

To provide a comprehensive understanding of broad-spectrum neuromodulation with the BPVE, ultraviolet photoelectron spectroscopy (UPS) is used to characterize the energy band profiles of the fabricated Te nanoflakes (Fig. 6c, d). The UPS analysis revealed that the Te exhibited a conduction band minimum of -4.73 eV, a valence band maximum of -5.07 eV, and a work function of -4.98 eV, confirming their classification as p-type semiconductors, which is caused by active vacancy defects^41,42^. When the Te are placed in the solution and in contact neurons, the energy band of the Te bends down^43^. Under light illumination, electrons in the valence band are stimulated to the conduction band and holes are created in the valence band, respectively. Photogenerated electrons move toward the surface of the Te due the to bending energy band (Fig. 6e and f). The accumulated electrons on the surface then spread into an electrolyte solution and diffused to the extracellular side of the neuronal membrane. The electrons diffused to the extracellular side and react with aqueous solution, leading to a change in the transmembrane voltage and subsequent depolarization of the neurons^44^. Photogenerated holes recombine within the Te nanoflake or solution. This process is also called photoelectrochemical reduction (Red) and oxidation (Ox).

## Discussion

In conclusion, we demonstrated a strong BPVE in Te and its application in broad-spectrum neuromodulation. The generated photocurrent in uniformly illuminated Te, excluding other photoelectric effects, is attributed to the BPVE. The bulk photovoltaic wavelength of narrow bandgap Te covers a broad wavelength range from the ultraviolet to the mid-infrared, while the achieved photocurrent density of 70.4 A cm^−2^ under infrared light simulation surpasses the performance of previous semiconductors and semimetals. The discovered infrared BPVE provides a versatile platform for exploring optoelectronic applications. Furthermore, the broad-spectrum neuromodulation achieved through Te with infrared BPVE, especially encompassing the entire NIR-IIa region (1.3–1.4 μm) with deep penetration (Supplementary Table 4). The high conversion efficiency of infrared BPVE ensures that the maximal temperature changes of 0.18 °C do not pose a risk of damaging neurons or tissues. Te with infrared BPVE emerges as a promising candidate for novel nano-modulation and holds great potential for the treatment of neurological diseases.

## Materials and methods

### Material characterization

Raman spectra were obtained from confocal microscopy (HR800) with a 532 nm laser source. JEOL JEM-2100F transmission electron microscopy (TEM) with the acceleration voltage of 200 kV was used to acquire TEM and STEM characterization data of Te.

### Device fabrication

Te grown on SiO_2_/Si substrates were mechanically transferred to the Si substrates covered with 280 nm SiO_2_. The substrates were immersed in the acetone solution to obtain Te that were solidly attached to the substrate. Poly(methylmethacrylate) (PMMA) was spin-coated on the substrates. After standard electron-beam lithography, Te electrodes were patterned. Then, platinum (30 nm) and gold (200 nm) were sequentially deposited via double ion beam sputtering.

### Photoelectric response measurements

The SPM of Te devices were measured by confocal microscopy, using a preamplifier (Stanford Research Systems SR 570), and phase lock equipment (Stanford Research Systems SR 830). The lock-in frequency was 277.7 Hz. Diode lasers were focused by the objective lens with a ×100 magnification and NA value of 0.7. Spot diameters of 637 nm, 830 nm, 1.31 µm, and 1.55 µm laser were 1.0 µm, 1.2 µm, 1.5 µm, and 2 µm, respectively. The time-resolved photocurrent, *I*-*V* curves, and polarization-dependent photocurrent were measured via a ×20 magnification objective to ensure that the light could cover the whole Te device. These data were simultaneously acquired by a Keysight B2912A source. For the polarization measurements of Te devices, a λ/2 plate was used to modulate the orientation of the linear polarization light after a THORLABS polarizer, respectively.

### Mouse primary cortex neuron culture

All mice were maintained under standard housing conditions of 22 ± 1°C, 50 ± 10% relative humidity and a 12 h light-dark cycle with food and water. Animal protocols were approved by the Institutional Animal Care and Use Committees of ShanghaiTech University, China. The primary cortex neurons were taken randomly from the embryonic 18-day ICR mouse cerebral cortex. Firstly, cerebral cortex tissues were dissociated and digested for 30 min by 0.5% DNAse and 0.25% papain mixture at 37 °C. The solution was transferred into 15 mL centrifuge tubes. Then, a centrifugal with 1000 rpm for 4 min was used to collect the cortical tissues. The cortex tissue blocks were blown and transformed into a single cell in a neurobasal medium containing 10% fetal bovine serum (gibco), 2 mM Glutamine (gibco), and 1% Penicillin/streptomycin (gibco). The single-cell solution was filtered through a 40 micro cell strainer and plated at a density of 0.5 × 10^6^ cells/mL on Polylysine (0.2 mg/ml)-treated 24 well plates in Neurobasal (gibco) medium with 10% fetal bovine serum (gibco), 2 mM Glutamine (gibco), and 1% Penicillin/streptomycin (gibco). After ~5 h, cells were cultured in serum-free Neurobasal medium with 2% B27 (gibco), 2 mM Glutamine (gibco), and 1% Penicillin/streptomycin mixture (gibco). Half of the media was replaced by fresh feeding media every 3 days. Finally, Te nanoflakes were added to the media. The concentration of Te nanoflakes is less than 5 µg/mL. Neurons and Te nanoflakes were co-cultured for 24 h before the electrophysiological recordings.

### Electrophysiological recordings

Whole-cell patch-clamp recordings of DIV11 mouse primary cortex neurons were recorded by an internal solution (136 mM K-gluconate, 6 mM KCl, 1 mM EGTA, 2.5 Na2ATP, 10 mM HEPES (280 mOsm, pH=7.2 with KOH)) and a cell-extracellular solution ACSF (126 mM NaCl, 4.9 mM KCL, 1.2 mM KH_2_PO_4_, 2.4 mM MgSO_4_, 2.5 mM CaCl_2_, 26 mM NaHCO_3_, 20 mM Glucose). The resistance of recording pipettes was 10-12 MΩ. 637 nm, 940 nm, and 1.31 µm lasers were focused and illuminated on Te nanoflakes adhered to the neurons by a 60× objective. The cultured cortical neurons were randomly allocated into recordings for different experimental groups. The corresponding diameters of 637 nm, 940 nm, and 1.31 μm light in our experiment were obtained to be 19.8 µm, 45.2 µm, and 74.6 µm by the Thorlabs Beam Profilers, respectively. Action potential data were collected with a 2 kHz low-pass filter (Multiclamp 700B and Digidata 1322 A/D converter) and sampled by a 10 kHz. Action potential characteristics were analyzed by using CLAMPFIT10.7 software. Data were collected and analyzed blindly by experimenters. All attempts at replicate were successful. P values were determined by Student’s t test.

### Immunohistochemistry

For immunohistochemistry, neurons were fixed with 4% paraformaldehyde (PFA) for 4 h at 4 °C. After fixation, the neurons were washed three times with 0.1 M PBS (pH 7.4) at room temperature. Subsequently, the neurons were blocked with a solution containing 10% goat serum, 3% BSA, and 0.3% Triton X-100 in 0.1% PBS for 2 h at room temperature. The neurons were then incubated overnight at 4 °C with the primary antibody solution, including rabbit anti-Cleaved-Caspase-3 (Abcam ab2302; 1:1000), mouse anti-NeuN (Abcam ab104224; 1:1000), rabbit anti-NeuN (Abcam ab177487; 1:1000), mouse anti-oligo2 (Abcam ab109186; 1:800), and mouse anti-S100β (Abcam ab41548; 1:800). Following incubation, the neurons were washed three times with PBS containing 0.1% Triton X-100 and stained with goat anti-rabbit Alexa 488 (Invitrogen A11008; 1:1000) and goat anti-mouse Alexa 546 (Invitrogen A11003; 1:1000) for 2 h at room temperature. DAPI was added for counterstaining, and the neurons were mounted onto slides. Images were acquired using a Zeiss 980 confocal microscope and processed using ImageJ.

### Temperature measurements

Thermometer pipettes with resistances of 3 MΩ were filled with bath solution and positioned 2 μm from the neuron/Te interface under investigation. The resistance of the pipette was tracked as part of a voltage divider circuit using a voltage amplifier while action potentials were generated in the adjacent cell. To convert pipette resistance to temperature, a calibration curve was individually created for each pipette by correlating resistance values with a wide range of temperatures, starting from 32.5 °C and allowing the solution to cool passively to room temperature. During this calibration process, a thermocouple positioned very close to the pipette tip simultaneously recorded the temperature.

## Supplementary information

Supplementary Information for Giant infrared bulk photovoltaic LIGH-spectrum neuromodulation

## Data Availability

The Source data underlying the figures of this study are available with the paper. Data that support the findings of this study are available from the corresponding authors upon reasonable request. Source data are provided with this paper.
