# Supplementary material for: Giant infrared bulk photovoltaic effect in tellurene for broad-spectrum neuromodulation
Source: Light Sci Appl. 2024 Sep 27;13:277. doi: 10.1038/s41377-024-01640-w (PMC11427709; doi:10.1038/s41377-024-01640-w)
Supplement: Supplementary file 1 — Supplementary Information for Giant infrared bulk photovoltaic LIGH-spectrum neuromodulation [file 41377_2024_1640_MOESM1_ESM.pdf]

Supplementary Information

Giant infrared bulk photovoltaic effect in tellurene for broad-spectrum neuromodulation

Zhen Wang, Chunhua Tan, Meng Peng, Yiye Yu, Fang Zhong, Peng Wang, Ting He, Yang Wang, Zhenhan Zhang, Runzhang Xie, Fang Wang, Shuijin He, Peng Zhou, and Weida Hu

Table of contents

Supplementary Note 1: Growth and characterizations of Te.. 4
Supplementary Fig. 1 | Growth setup schematic and corresponding images of CVD-based Te 5
Supplementary Fig. 2 | X-ray diffraction pattern and absorption spectrum of Te nanoflakes... 5
Supplementary Fig. 3 | Surface quality of Te nanoflake. 6
Supplementary Fig. 4 | TEM and STEM characterizations of synthesized Te.......................... 7
Supplementary Fig. 5 | Morphology and length of Te via different growth temperatures. 7
Supplementary Note 2: Fabrication and photoelectric response of Te devices 7
Supplementary Fig. 6 | Photoelectric response in Te device #1 9
Supplementary Fig. 7 | Photoelectric response in Te device #2 10
Supplementary Fig. 8 | Photoelectric response in Te NS device #1 10
Supplementary Fig. 9 | Photoelectric response in Te device #2 11
Supplementary Note 3: Overview and distinction of various photoelectric effects 11
Supplementary Table 1 | Overview of photoelectric effects from the view of SPM characterization and optoelectrical properties 14
Supplementary Note 4: Influence of oblique incidence on the Te 16
Supplementary Fig. 10 | Photovoltaic response in Te device #2 induced by oblique incident laser light 16
Supplementary Fig. 11 | Photocurrent profiles of Te under light illumination........................ 17
Supplementary Fig. 12 | BPVE in Te device #3.. 18
Supplementary Fig. 13 | BPVE in Te device #4.. 19
Supplementary Fig. 14 | BPVE in Te device #5.. 20
Supplementary Fig. 15 | BPVE in Te device #6.. 21
Supplementary Fig. 16 | BPVE in Te device #7.. 22
Supplementary Fig. 17 | BPVE in Te device #8.. 23
Supplementary Table 2 | Photocurrent of Te nanoflakes as the relationship of the size 24
Supplementary Note 5: Overview of the BPVE in various materials. 24
Supplementary Table 3 | Comparison of maximum response wavelength and photocurrent density among previous materials with the BPVE. 26
Supplementary Fig. 18 | Proportion of oligodendrocytes, astrocytes, and neurons................. 27
Supplementary Fig. 19 | Summary of microscopy images of the recorded neurons that are in contact with Te nanoflakes in response to 637 nm light. 28
Supplementary Fig. 20 | Summary of microscopy images of the recorded neurons with Te .. 29
Supplementary Fig. 21 | Summary of microscopy images of the recorded neurons with Te

nanoflakes for 1.31 μm light. 30
Supplementary Note 6: Degradation of Te. 30
Supplementary Fig. 22 | Te in ACSF solution during different days 31
Supplementary Fig. 23 | Characterizations of Te nanoflakes in ACSF solution 32
Supplementary Fig. 24 | Summary of microscopy images of the recorded neurons without Te. Neurons were cultured in the medium containing no Te nanoflakes. 33
Supplementary Note 7: ACS solution pH and condition of neurons after long illumination cycles. 33
Supplementary Fig. 25 | The pH changes of the ACSF solution under 1.31 μm light illumination were recorded over time. 34
Supplementary Fig. 26 | No apoptosis was observed in neurons co-cultured with Te nanoflakes under 1.31 μm light illumination at various time intervals. 35
Supplementary Fig. 27 | Resting membrane potential of neurons was measured under 637 nm, 940 nm, and 1.31 μm light illumination for 5 minutes. 35
Supplementary Fig. 28 | Photocurrent signal diagram of Te-based patch-clamp recording.... 36
Supplementary Note 8: Diameters and power densities of light with different wavelengths.. 36
Supplementary Fig. 29 | Spot size of light with different wavelengths. 37
Supplementary Fig. 30 | Action potentials are not generated in the neurons without Te under 637 nm light illumination. 38
Supplementary Fig. 31 | Action potentials are not generated in the neurons without Te under 940 nm light illumination. 38
Supplementary Fig. 32 | Action potentials are not evoked in the neurons without Te under 1.31 μm light illumination. 39
Supplementary Fig. 33 | Neuromodulation of mouse primary cortical neurons with Te under 940 nm light irradiation 39
Supplementary Fig. 34 | Neuromodulation of mouse primary cortical neurons with Te under 637 nm light irradiation 40
Supplementary Fig. 35 | Action potentials are elicited in the neurons with Te under 1.55 μm light irradiation. 41
Supplementary Fig. 36 |Action potentials are evoked in the neurons with Te under 637 nm light irradiation. 42
Supplementary Fig. 37 | Action potentials are elicited in the neurons with Te under 940 nm light irradiation. 42
Supplementary Fig. 38 | Action potentials are evoked in the neurons with Te under 1.31 nm light irradiation. 43
Supplementary Fig. 39 | Factors of Te-based neuromodulation. 43
Supplementary Note 9: Photocurrent and photovoltage generated by the Te nanoflakes using the patch clamp setup. 44
Supplementary Fig. 40 | Generated photocurrent of a Te nanoflake in optical neuromodulation. 45
Supplementary Fig. 41 | Photocurrent of Te nanoflake related to 1.31 μm light pulses. 45
Supplementary Fig. 42 | Photovoltage generated by the Te nanoflake using the patch clamp setup in the current clamp 46
Supplementary Note 10: Temperature changes of Te under light irradiation. 46

Supplementary Fig. 43 | Temperature changes of Te under light irradiation. 47
Supplementary Fig. 44 | Schematic of temperature measurement setup for simultaneous measurement of temperature and neuronal action potentials produced by laser stimulation. . 48
Supplementary Fig. 45 | Temperature measurement of the neuron and Te nanoflake. 48
Supplementary Table 4 | Comparison of photoelectric neuromodulation 40

Supplementary Notes

Supplementary Note 1: Growth and characterizations of Te.

Te nanoflakes were synthesized on SiO_2/Si substrates using chemical vapor deposition (CVD). The growth process, as illustrated in Supplementary Fig. 1a, consisted of three main components: gas intake, reactor, and vacuum systems. Initially, a ceramic boat containing tin telluride ($SnTe_2$) powder (Aldrich, 99.99% purity) was placed at the center of a quartz tube. A 280 nm SiO_2/Si substrate was positioned on a quartz boat downstream in the quartz tube, as shown in Supplementary Figs. 1b-d. Subsequently, a pumping system, comprising a mechanical pump and control valves, was activated to evacuate the air from the quartz tube. The pressure was reduced to below 10 Pa for approximately 30 minutes. A gas intake subsystem, equipped with a mass flow controller (MFC), regulated the flow of nitrogen gas (N_2) at a rate of 50 standard cubic centimeters per minute (sccm). The reactor system employed a Thermo Scientific Lindberg/Blue M TF55035C-1 furnace. The $SnTe_2$ source was heated to 650 °C and maintained at that temperature for 30 minutes. After natural cooling, the SiO_2/Si substrate was retrieved, and Te were synthesized on the substrate.

The morphology and size of Te play a crucial role in their application for optical neuromodulation. In particular, the length of Te needs to be much smaller than that of neurons. To control the length of Te, we found a close relationship between the growth process of Te, atomic migration rate, and temperature. The relationship can be described by the Arrhenius equation: $D = D_0 \times \exp(-Q/kT)$. D represents the atomic migration rate. D_0 is the pre-exponential factor or pre-factor. Q is the activation energy associated with the migration process. k and T represent the Boltzmann constant and the temperature, respectively.

According to the Arrhenius equation, as the temperature increases, the atomic migration rate exponentially increases. The temperature rise enhances the thermal energy of atoms or ions, enabling them to overcome activation energy or potential barriers and accelerate the growth rate of Te. By regulating the temperature of the growth region on the substrate, it is possible to achieve a small and relatively constant length of Te. The $SnTe_2$ source temperature in the furnace is approximately 650 °C.

When the temperature of the growth region on the substrate increases from 200 °C to 400 °C, the length of Te changes from 0.95 μm to 12.92 μm, as shown in Supplementary Fig. 5. The longest length of Te exceeds 20 μm. However, as the source temperature increases up to 650 °C, the size of Te decreases to 5 μm due to the crystal nucleation and growth rate[1].

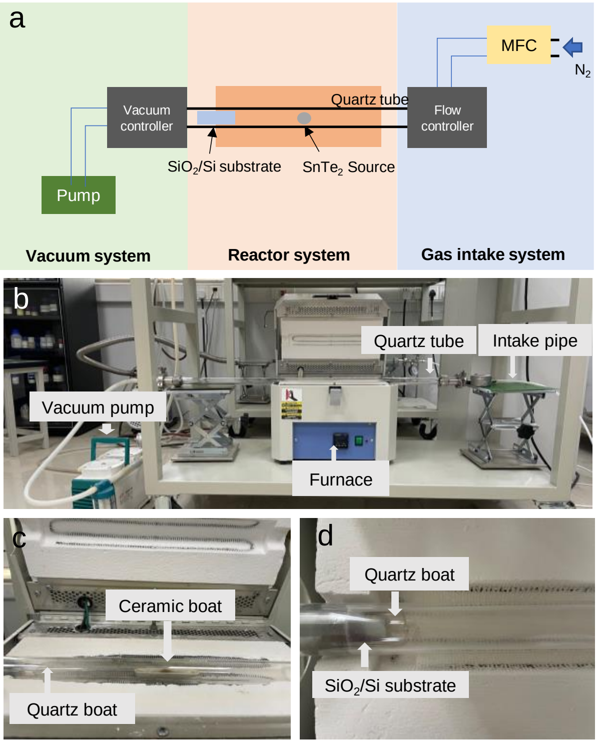

Supplementary Fig. 1 | Growth setup schematic and corresponding images of CVD-based Te.

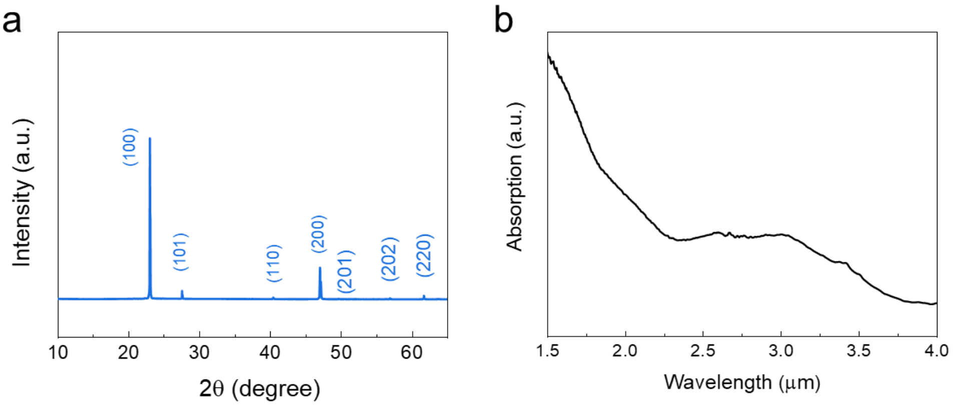

Supplementary Fig. 2 | X-ray diffraction pattern and absorption spectrum of Te

nanoflakes.

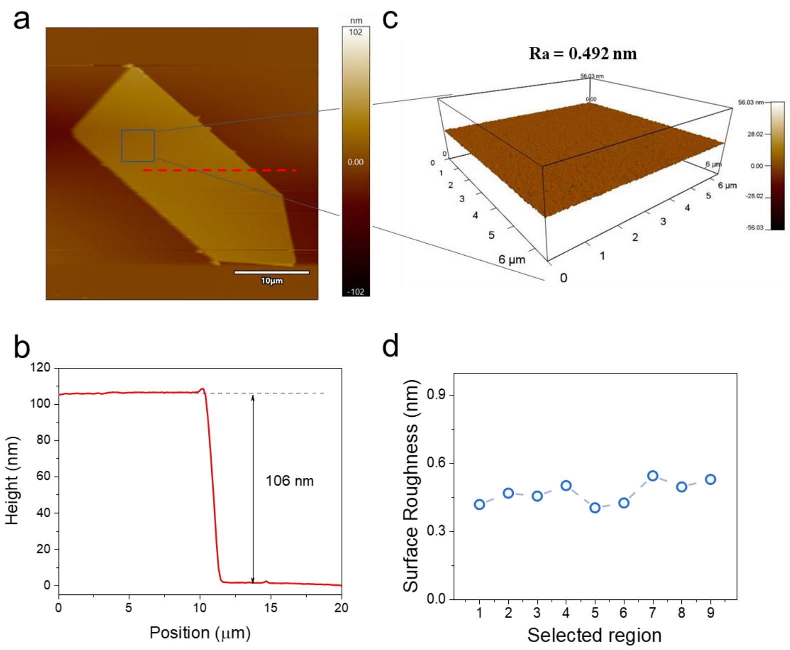

Supplementary Fig. 3 | Surface quality of Te nanoflake. a, Atomic force microscopy image of Te nanoflake. **b,** Height profile of Te nanoflake along the red line in Figure a. **c,** Enlarged surface microscopy image of Te nanoflake. The area is approximately 6 μm × 6 μm. **d,** Surface roughness of Te nanoflake obtained 9 regions (6 μm × 6 μm). The height of Te nanoflake is approximately 106 nm. The surface roughness of Te nanoflake obtained 6 μm × 6 μm area is about 0.492 nm. The average surface roughness of Te nanoflake is less than 0.5 nm, indicating a high surface quality.

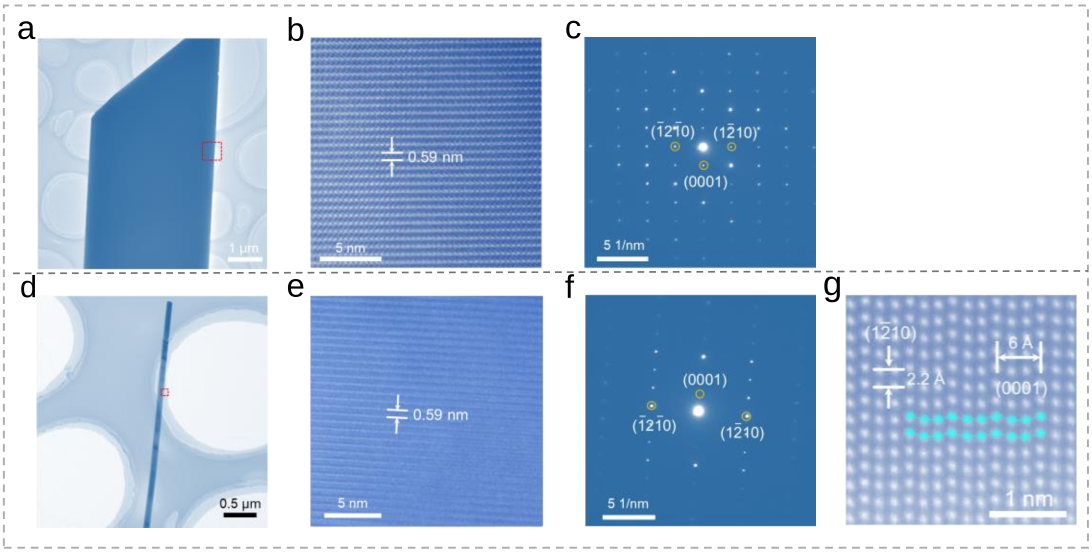

Supplementary Fig. 4 | TEM and STEM characterizations of synthesized Te. a, b, and **c,** Low-magnification, high-magnification TEM, and SAED images of the Te with a width great than 1 μm. **d, e, f,** and **g,** Low-magnification, high-magnification TEM, SAED, and STEM images of the Te with a width less than 1 μm. The interplanar distance of 0.59 nm is the crystal spacing distance of the (0001) plane, indicating that the crystallized Te grow along with the [0001] orientation.

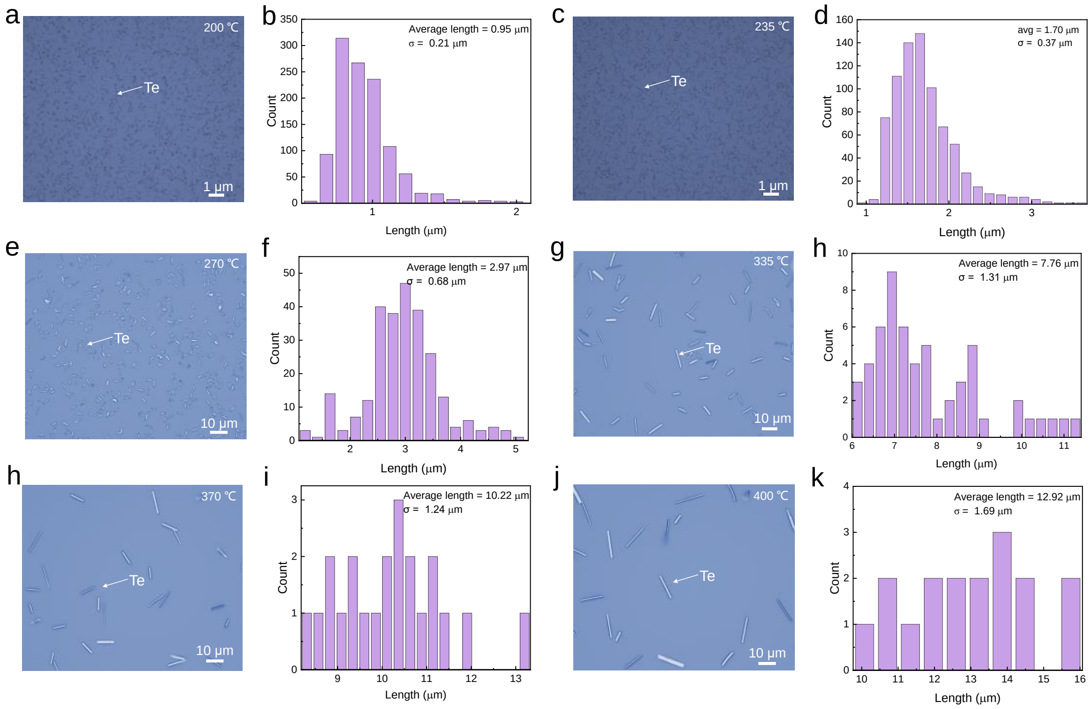

Supplementary Fig. 5 | Morphology and length of Te via different growth temperatures. a, c, e, g, and **h,** Optical microscope images of Te by changing growth temperature. **b, d, f, h,** and **i** Length distribution of Te, which are extracted from corresponding optical images. σ represent the standard deviation of the length, respectively.

Supplementary Note 2: Fabrication and photoelectric response of Te devices

Due to the high work function of platinum, platinum and subsequent gold are selected as the contact metals[2]. Supplementary Figures 6a-6c show the SPM of Te device #1 under 637 nm, 830 nm, and 2 μm laser illumination. Large I_{ph} generates among the Te channel rather than the contact interfaces between the Te and contact

electrodes. The I_{ph} profiles in Supplementary Figs. 6d-6f further demonstrate that the larger I_{ph} generates among the Te channel. Meanwhile, the negative I_{ph} is generated at the electrode under 637 nm, 830 nm, and 2 μm laser illumination. The I_{ph} becomes positive under 2 μm laser illumination. However, the I_{ph} generated in channel Te is much bigger than the negative one in the interfaces, indicating that the intrinsic response in Te channel is dominant.

The SPM of Te device #2 are shown in Supplementary Figs. 7a and 7b. There is a similar trend that I_{ph} generates among the Te channel under 637 nm and 1.55 μm laser illumination. Compared to the Te device #1, no negative I_{ph} is observed in the extracted I_{ph} profiles, as shown in Supplementary Fig. 7c and 7d.

Time-resolved I_{ph} of Te devices were obtained at zero voltage. The spot size of the lasers with different wavelengths is far larger than the size of Te devices to ensure uniform illumination. Time-resolved I_{ph} of Te device #1 under 637 nm, 830 nm, and 1.55 μm laser illumination are shown in Supplementary Figs. 8a, 8b, and 8c, respectively. When the lasers are turned on, the photocurrent is quickly generated. In dark condition, the *I-V* curve of Te device #1 pass through the origin, shown in Supplementary Fig. 8d. When the laser illumination is on and the power increases, short-circuit current and open-circuit voltage appear and increase. This kind of nonzero characteristic is one of the photovoltaic effects.

Time-resolved I_{ph} of Te device #2 under 830 nm, 1.31 μm, and 1.55 μm laser illumination are shown in Supplementary Figs. 9a-9c. An obvious photocurrent in the Te device #1 generates. Supplementary Figure 9d shows the I_{ph} versus laser power density in Te device #2 for infrared wavelength. As the power increases, the photoelectric response in the Te device #2 becomes strong. The power dependence also reveals a transition from a linear to a sub-linear dependence, which is similar to the previous work[6].

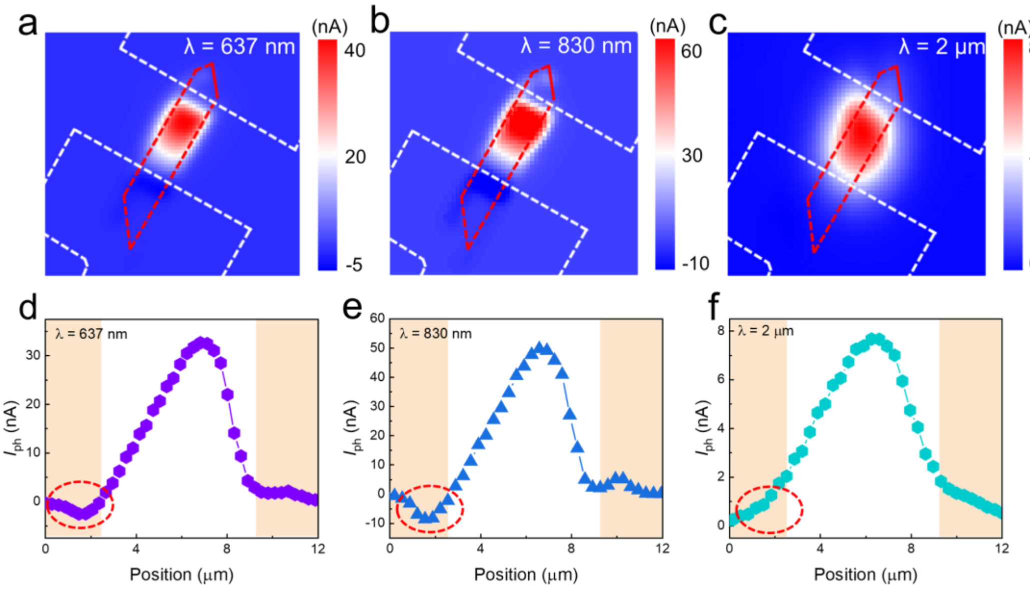

Supplementary Fig. 6 | Photoelectric response in Te device #1. a-c, SPM of Te device #1 under 637 nm, 830 nm, and 2 μm laser illumination. The applied voltage is zero. Shapes or boundaries of low-dimensional Te and contact electrodes are outlined in dashed red and white lines, respectively. The shape of Te NS device #1 is pentagonal. **d-f,** I_{ph} profiles extracted along the center lines of the Te NS channel in **a**-**c**. Power densities of 637 nm, 830 nm, and 2 μm light are 5.43, 0.24, and 0.021 mW mm^{-2}, respectively.

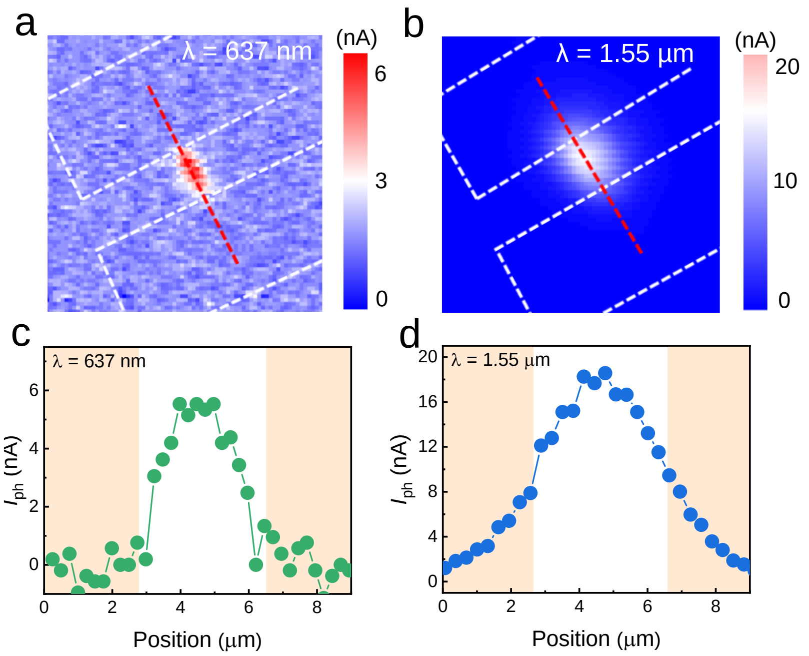

Supplementary Fig. 7 | Photoelectric response in Te device #2. a-b, SPM of Te device #2 under 637nm and 1.55 μm laser illumination. The applied voltage is zero. Shapes or boundaries of low-dimensional Te and contact electrodes are outlined in dashed red and white lines, respectively. **c-d,** I_{ph} profiles extracted along the center lines of the Te channel in **a**-**b**. Power densities of 637 nm and 1.55 μm light are 6.43 and 1.24 mW mm^{-2}, respectively.

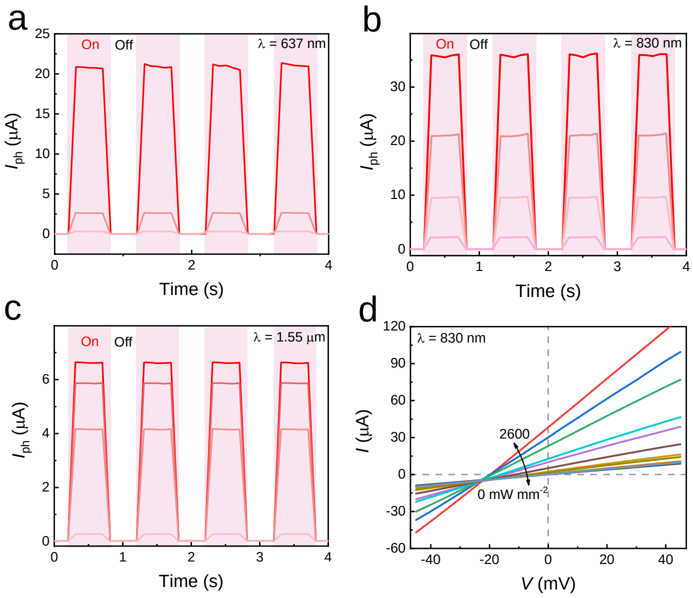

Supplementary Fig. 8 | Photoelectric response in Te NS device #1. a-c, Time-resolved I_{ph} of Te device #1 under 637 nm, 830 nm, and 1.55 μm laser illumination with different power densities. The power densities of 637 nm light are 3160, 1900, 178, and 17.8 mW mm^{-2}, respectively. The power densities of 830 nm light are 2320, 1890, 620, and 101 mW mm^{-2}, respectively. The power densities of 1.55 μm light are 99.5, 85.6, 54, and 2.4 mW mm^{-2}, respectively. **d,** I-V curves of Te device #1 under 830 nm laser illumination with the power density changing from 0 to 2600 mW mm^{-2}.

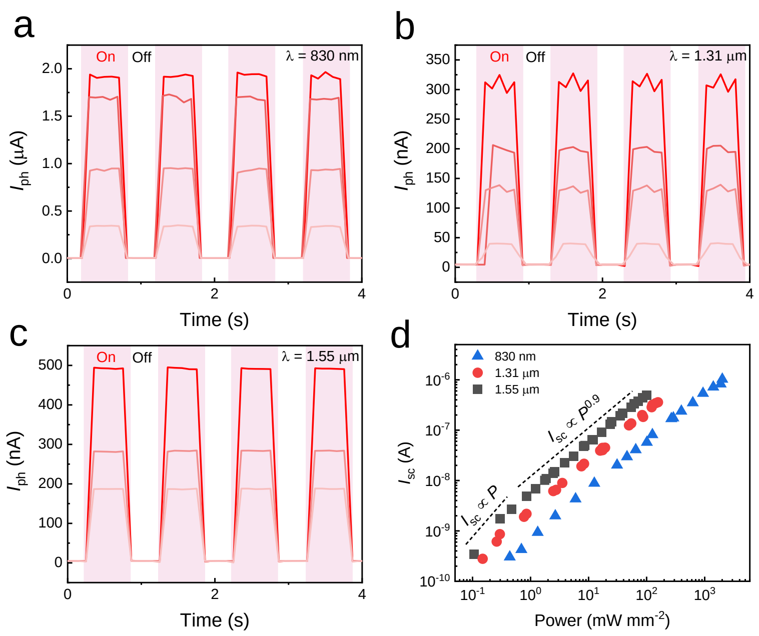

Supplementary Fig. 9 | Photoelectric response in Te device #2. a-c, Time-resolved I_{ph} of Te device #2 under 830 nm, 1.31 μm, and 1.55 μm laser illumination with different power densities. The power densities of 830 nm light are 2600, 2330, 1890, and 626 mW mm^{-2}, respectively. The power densities of 1.31 μm light are 125, 83, 54, and 17 mW mm^{-2}, respectively. The power densities of 1.55 μm light are 99.5, 85.5, and 35.1 mW mm^{-2}, respectively. **d,** I_{sc} versus laser power density in Te device #2 for infrared wavelength.

Supplementary Note 3: Overview and distinction of various photoelectric effects

Various photoelectric effects are intriguing and fundamental elements of modern optoelectronics. However, these photoelectric effect mechanisms are very complicated. Here, we summarize various photoelectric effects and distinguish them from the respective of device structure, *I-V* curves, scanning photocurrent mapping, and photoresponse-dependent bandgap, shown in Supplementary Table 1.

The photoconductive effect is based on a single and homogeneous semiconductor. The corresponding device is usually comprised of a semiconductor and two contact electrodes. The electrodes are always the same metal. Under light illumination, the free carrier in the semiconductor increases. Importantly, these photogenerated excess carriers need to be driven under the externally applied voltage and finally collected by

the metal electrodes. Normally, the generated photocurrent is larger than the dark current. However, the negative photocurrent is also observed due to the normal capture of electrons or holes[7,8]. SPM characterization result shows that the opposite photocurrent is detected near the metal-to-semiconductor interfaces under the applied voltage of zero. Moreover, the generated photocurrent is only observed within the narrow regions (less than 1 μm width)[3]. This kind of photoelectric effect is closely related to the bandgap of semiconductors because the photogenerated electrons or holes can't be inspired by the photon whose energy is less than the bandgap of the semiconductors.

Device configuration of photovoltaic effect is usually p-n junction. A p-type semiconductor abuts an n-type semiconductor. The photogenerated electron-hole pairs are separated by the built-in field in the p-n junction. The dark I-V curve is exponential. Under light illumination, non-zero open-circuit voltage and short-circuit current appear. The negative current mainly distributes among the p-n junction region. Additionally, the photoresponse wavelength lies on the bandgap of p-type or n-type semiconductors.

The BPVE appears in single crystals with broken inversion symmetry and does not require a p-n junction. This broken inversion symmetry can be realized by the materials with the polar space point group[9], a reduced-dimensional nanotube[10], and a van der Waals interface[6]. Different from the photovoltaic effect based on p-n junctions, the dark I-V curve of the BPVE is linear. When illuminated by the light, a non-zero curve characteristic is exhibited. The photocurrent generated among the whole channel is not the opposite. Meanwhile, the photocurrent disappears when the photon energy is smaller than the bandgap of crystals. As-grown Te is a homogeneously elemental crystal. Te belongs to a space point group with a noncentrosymmetry. Furthermore, the measured photoelectric characteristic of Te and devices is similar to the above-mentioned curves. Therefore, the observed photovoltaic in Te is caused by the BPVE.

The photothermoelectric effect is the thermal effect. As the spot of the illuminated light is smaller than the size of the device channel, this local light leads to a temperature gradient at the channel. This temperature difference between the different parts of the channel or the materials-to-metal interface gives rise to the thermoelectric current and

voltage. Aside from the local light illumination, the temperature gradient is produced by globe light when the light absorption of different parts of the channel varies. Both dark and illuminated *I-V* curves for the photothermoelectric effect are linear. It is worthy that SPM indicates that the opposite current generates in the entire channel with a homogeneous material and changes from the positive to the negative[3]. Remarkably, the photoresponse does not depend on the bandgap of the materials.

The flexo-photovoltaic effect is the coupling effect of electric polarization and strain gradient. The applied strain is key and makes a centrosymmetric semiconductor change into a non-centrosymmetric semiconductor. Photoelectric and SPM characteristics of flexo-photovoltaic are similar to that of the BPVE.

The photo-Dember effect is attributed to a larger mobility difference between electron and hole in a material. Under light illumination, the transport distance difference of electron and hole results in the generation of photocurrent. However, this kind of photocurrent usually depends on the broken symmetry from the surface or the material-to-metal contact. For the metal contact, the photocurrent produces in a very narrow region adjacent to the contact. The region width is approximately several hundred nanometers (the carrier mean free path)[11], which is very different from that of the photovoltaic response of Te. For the inhomogeneous sample, the photocurrent profile from SPM characterization reveals that the photocurrent should fluctuate around zero[10], which is also inconsistent with that of Te devices. Additionally, the photo-Dember effect is very weak in narrow bandgap semiconductors. Therefore, the photo-Dember effect is not responsible for the photovoltaic response in Te.

Photogalvanic and photon drag effects are that the photocurrent produces under an oblique incident laser light. SPM and *I-V* curves in Supplementary Table 1 are obtained from WSe_2 device[12]. Photogalvanic effect-based photocurrent is closely related to circular or linear polarization light. The influence of photogalvanic and photon drag effects on the Te will be discussed in Supplementary Note 4. As a result, the contribution of photogalvanic and photon drag effect is very small for the photovoltaic response in the Te.

The bolometric effect is that the resistivity of a temperature-sensitive material

increases or decreases when the materials are illuminated by light. The photocurrent is only observed by applying an external bias and does not rely on the wavelength of incident light.

In summary, the observed photovoltaic response in the Te is caused by the BPVE.

Supplementary Table 1 | Overview of photoelectric effects from the view of SPM characterization and optoelectrical properties. Red and blue regions represent the positive and negative photocurrent, respectively.

Photoelectrical effect	Device structure	*I-V* curve	SPM at zero bias	Photocurrent profile extracted from photocurrent mapping	Photoresponse-dependent material bandgap
Photoconductive effect	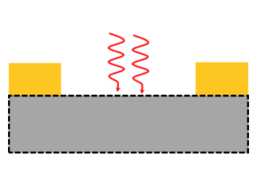	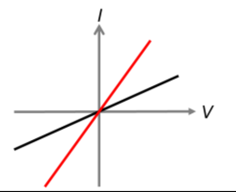	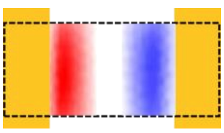	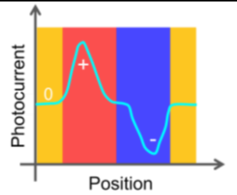	Yes
Photovoltaic effect	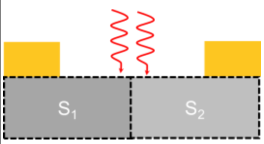	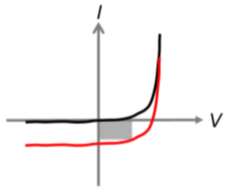	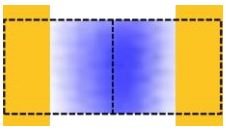	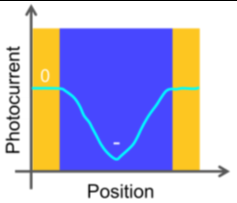	Yes
Bulk photovoltaic effect	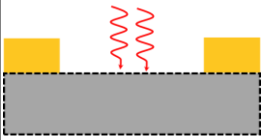	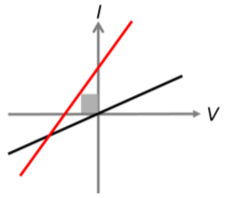	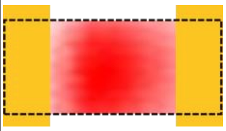	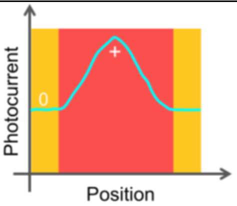	Yes
Photothermoelectric effect	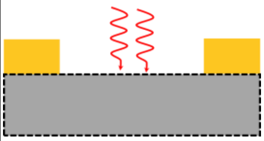	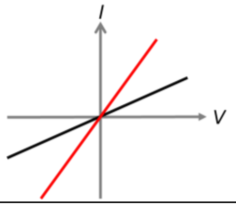	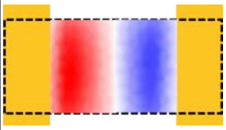	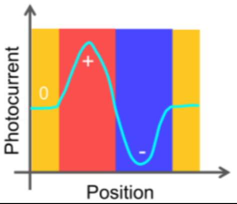	No
Flexo-photovoltaic effect	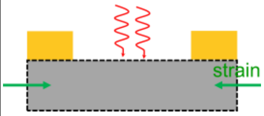	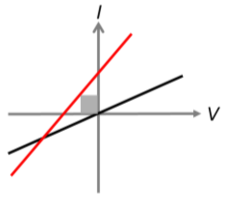	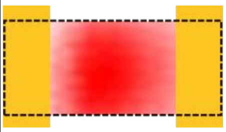	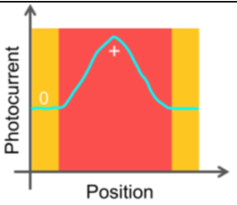	Yes
Photo-Dember effect	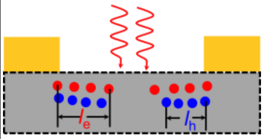		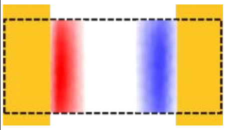	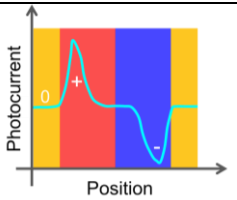	Yes
Photogalvanic and photon drag effect	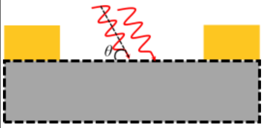		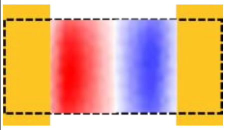	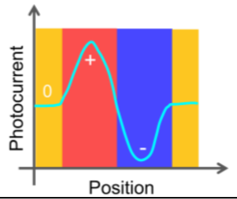	Yes
Bolometric effect	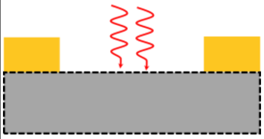	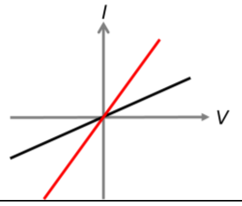			No

Supplementary Note 4: Influence of oblique incidence on the Te

Photogalvanic and photon drag effects are that the photocurrent produces under an oblique incident laser light. When laser light illuminates on the Te with the width of less than 1 μm, an incident angle between the light and the surface of the Te inevitably occurs. This oblique incident light also promotes the generation of the photocurrent in the Te, which is similar to the I_{ph} in the Te device #2 induced by the BPVE. However the light polarization θ dependence of the photocurrent can be used to distinguish the photogalvanic and the photon drag effects. Firstly, the incident laser is linearly polarized through a polarizer. Then a λ/4 plate is added to change the polarization to realize a circularly polarized light. The θ represents the angle between the fast axis of the λ/4 plate and the electric field direction of the linearly polarized light. The polarization dependence of I_{sc} in the Te device #1 is shown in Supplementary Fig. 10. The obtained data can be fitted by the following expression:

$$I_{sc}(\theta) = A\sin(2\theta + \theta_0) + L_1\cos(4\theta + \theta_0) + L_2\cos(4\theta + \theta_0) + B$$

Herein, A represents the amplitude of the circular photogalvanic and photon drag effects. L_1 is the magnitude of the linear photogalvanic effect. L_2 is the total of this oscillatory contribution of the BPVE and the linear photon drag effect. B represents the average value of the BPVE. After fitting, the obtained parameters are A = 2.77 nA, θ_0 = 3.04 rad, L_1 = -2 nA, L_2 = 24 nA, and B = 25.09 nA, respectively. Since the value of B is much bigger than that of A and L_1, the contribution of the BPVE to I_{sc} in Te device #2 is much larger than that from the photogalvanic and photon drag effects.

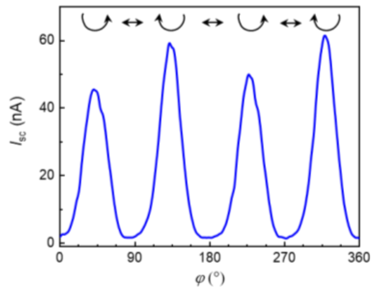

Supplementary Fig. 10 | Photovoltaic response in Te device #2 induced by oblique incident laser light. Polarization-dependence of I_{sc} for Te devices #2 under 1.3 μm

laser illumination.

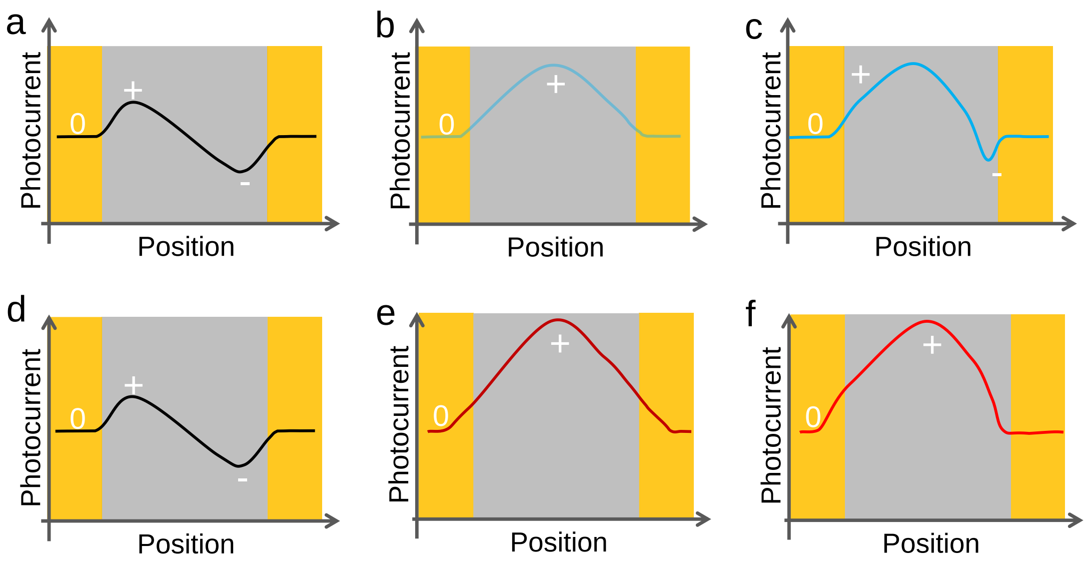

Supplementary Fig. 11 | Photocurrent profiles of Te under light illumination with different wavelengths. **a**, Photocurrent profile of Te caused by photothermoelectric effect under 637 nm, 830 nm, and 1.31 μm light illumination. **b**, Photocurrent profile of Te caused by the BPVE under 637 nm, 830 nm, and 1.31 μm light illumination. **c**, Photocurrent profile of Te caused by the BPVE and photothermoelectric effect under 637 nm, 830 nm, and 1.31 μm light illumination. **d,** Photocurrent profile of Te caused by photothermoelectric effect under 1.55 μm and 2 μm light illumination. **e,** Photocurrent profile of Te caused by the BPVE under 1.55 μm and 2 μm light illumination. **f,** Photocurrent profile of Te caused by the BPVE and photothermoelectric effect under 1.55 μm and 2 μm light illumination.

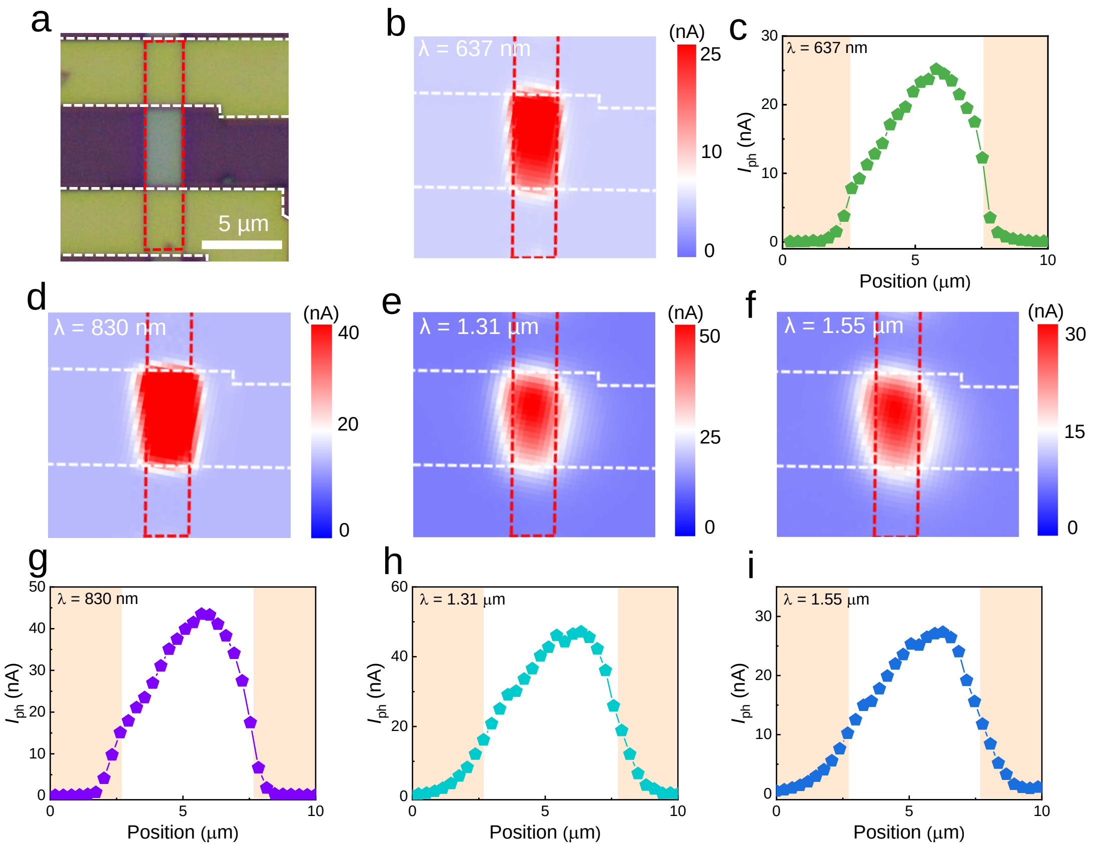

Supplementary Fig. 12 | BPVE in Te device #3. a, Optical microscopy image of Te device #3. **b, d-f,** SPM of Te device #3 under 637nm, 830 nm, 1.31 µm, and 1.55 µm laser illumination. The applied voltage is zero. Shapes or boundaries of the Te and contact electrodes are outlined in dashed red and white lines, respectively. **c, g-i,** I_{ph} profiles extracted along the center lines of the Te channel in **b, d-f**. Power densities of 637 nm, 830 nm, 1.31 µm and 1.55 µm light are 0.64, 0.095, 0.087, and 0.043 mW mm^{-2}, respectively.

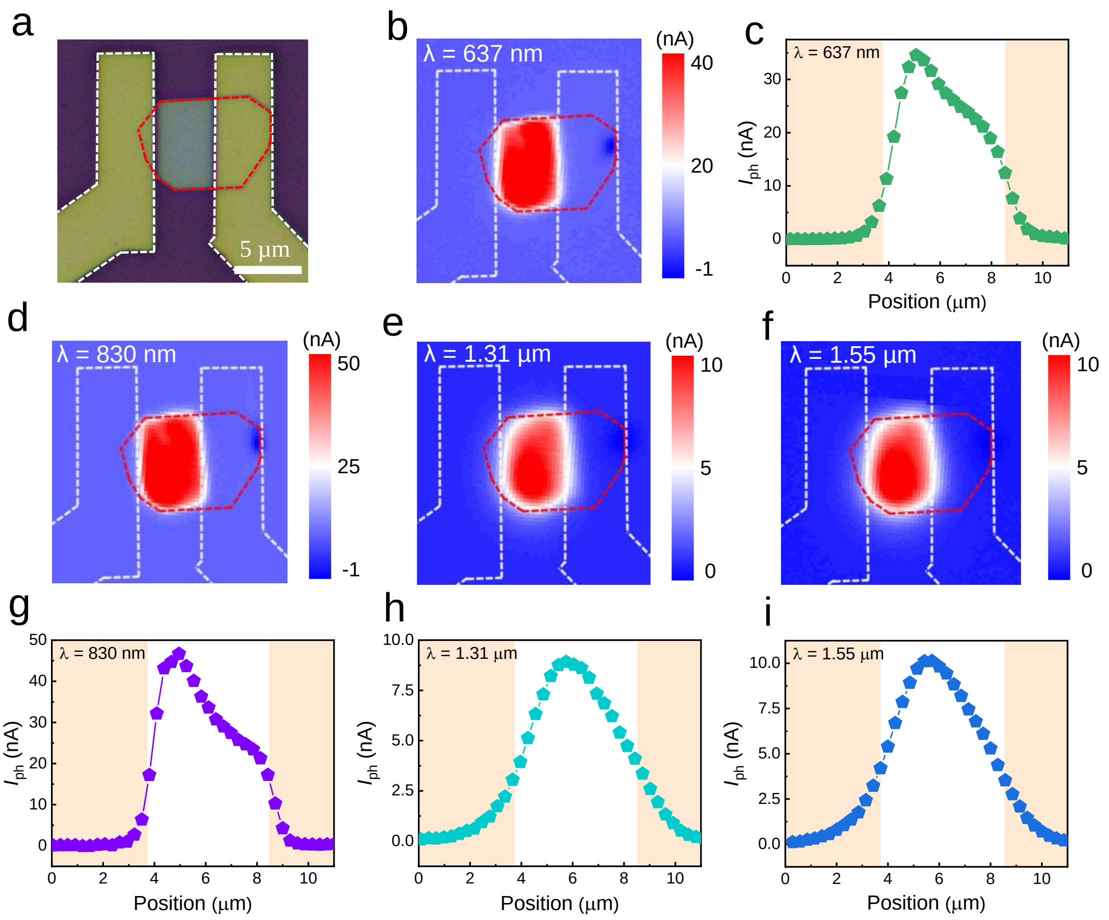

Supplementary Fig. 13 | BPVE in Te device #4. a, Optical microscopy image of Te device #4. The shape of Te device #4 is octagonal. **b, d-f,** SPM of Te device #4 under 637nm, 830 nm, 1.31 µm, and 1.55 µm laser illumination. The applied voltage is zero. Shapes or boundaries of the Te and contact electrodes are outlined in dashed red and white lines, respectively. **c, g-i,** I_{ph} profiles extracted along the center lines of the Te channel in **b, d-f**. Power densities of 637 nm, 830 nm, 1.31 µm and 1.55 µm light are 0.73, 0.085, 0.068, and 0.032 mW mm^{-2}, respectively.

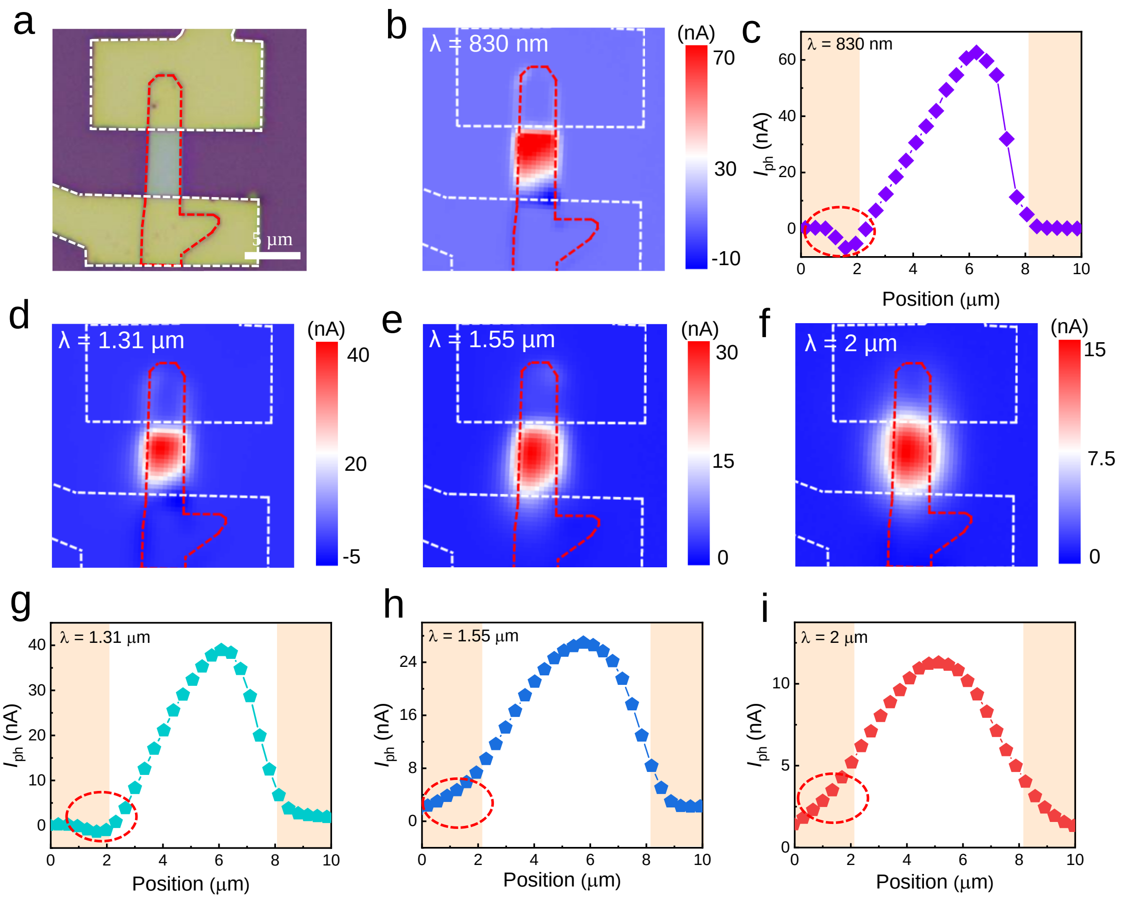

Supplementary Fig. 14 | BPVE in Te device #5. a, Optical microscopy image of Te device #5. **b, d-f,** SPM of Te device #5 under 830 nm, 1.31 μm, 1.55 μm, and 2 μm laser illumination. The applied voltage is zero. Shapes or boundaries of the Te and contact electrodes are outlined in dashed red and white lines, respectively. **c, g-i,** I_{ph} profiles extracted along the center lines of the Te channel in **b, d-f**. Power densities of 830 nm, 1.31 μm, 1.55 μm, and 2 μm light are 0.065, 0.078, 0.045, and 0.022 mW mm^{-2}, respectively.

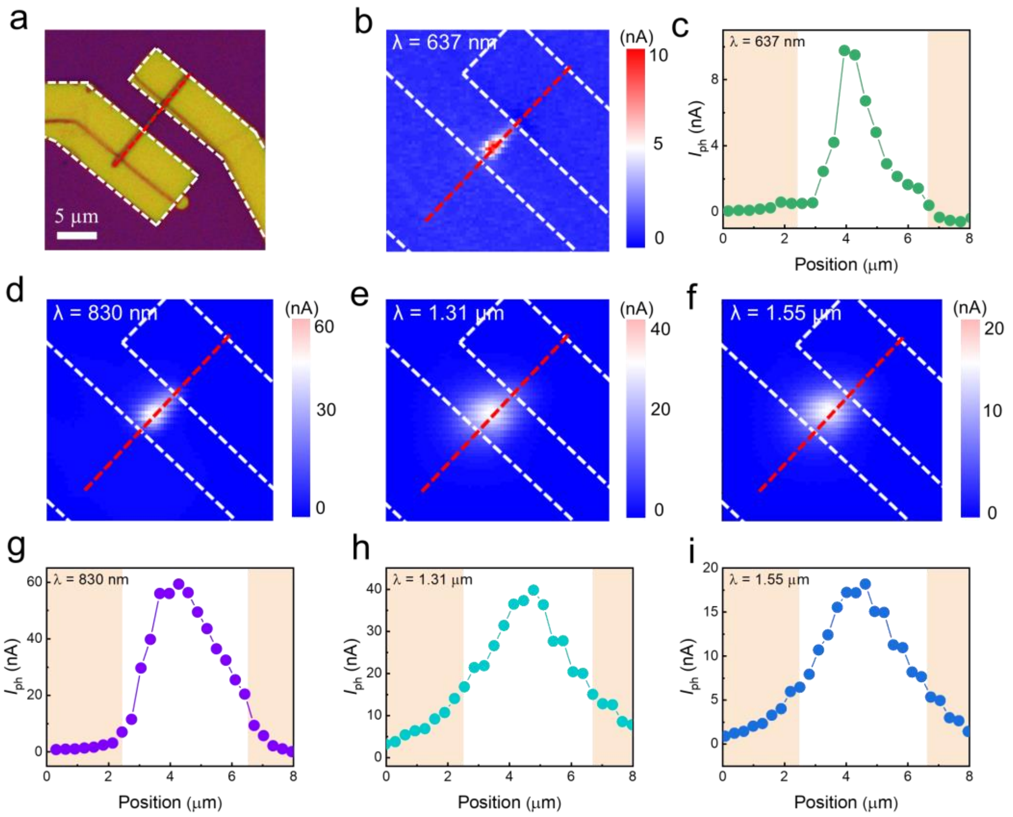

Supplementary Fig. 15 | BPVE in Te device #6. a, Optical microscopy image of Te device #6. **b, d-f,** SPM of Te device #6 under 637nm, 830 nm, 1.31 µm, and 1.55 µm laser illumination. The applied voltage is zero. Shapes or boundaries of the Te and contact electrodes are outlined in dashed red and white lines, respectively. **c, g-i,** I_{ph} profiles extracted along the center lines of the Te channel in **b, d-f**. Power densities of 637 nm, 830 nm, 1.31 µm, and 1.55 µm light are 5.73, 0.25, 0.28, and 0.12 mW mm^{-2}, respectively.

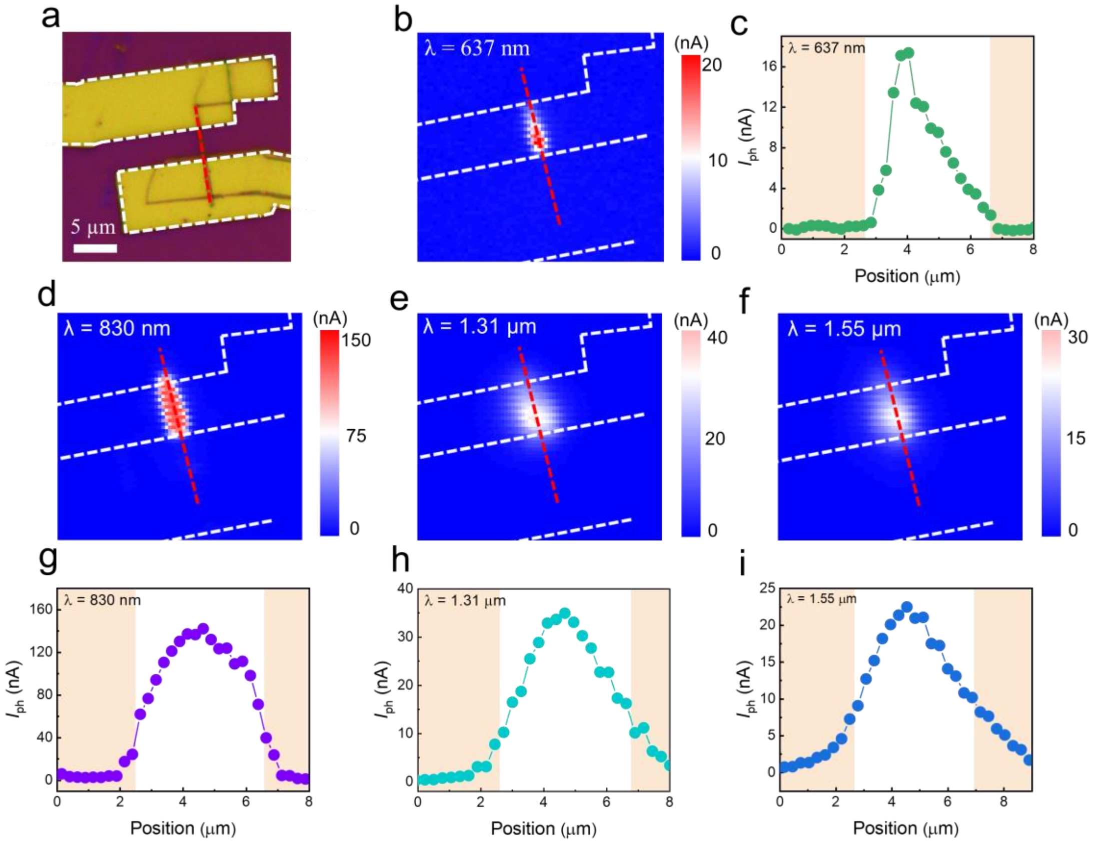

Supplementary Fig. 16 | BPVE in Te device #7. a, Optical microscopy image of Te device #7. **b, d-f,** SPM of Te device #7 under 637nm, 830 nm, 1.31 μm, and 1.55 μm laser illumination. The applied voltage is zero. Shapes or boundaries of the Te and contact electrodes are outlined in dashed red and white lines, respectively. **c, g-i,** I_{ph} profiles extracted along the center lines of the Te channel in **b, d-f**. Power densities of 637 nm, 830 nm, 1.31 μm, and 1.55 μm light are 2.73, 8.24, 10.01, and 9.78 mW mm^{-2}, respectively.

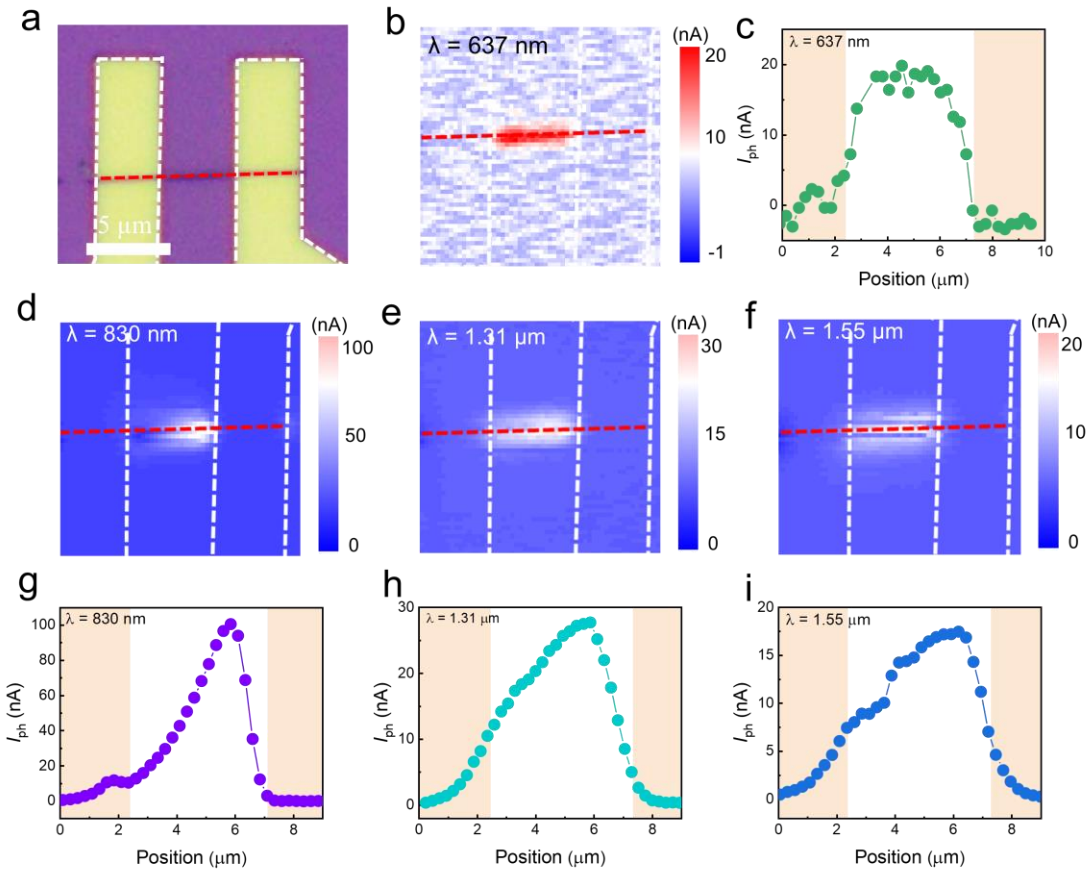

Supplementary Fig. 17 | BPVE in Te device #8. a, Optical microscopy image of Te device #8. **b, d-f,** SPM of Te device #8 under 637nm, 830 nm, 1.31 μm, and 1.55 μm laser illumination. The applied voltage is zero. Shapes or boundaries of the Te and contact electrodes are outlined in dashed red and white lines, respectively. **c, g-i,** I_{ph} profiles extracted along the center lines of the Te channel in **b, d-f**. Power densities of 637 nm, 830 nm, 1.31 μm and 1.55 μm light are 2.73, 10.25, 8.75, and 9.72 mW mm^{-2}, respectively.

Supplementary Table 2 | Photocurrent of Te nanoflakes as the relationship of the size.

Te	Length (μm)	Width (μm)	Light	Photocurrent (A)
Device#1	6.8	2.0	1.31 μm@ 3.52 mW mm^{-2}	3.99×10^{-7}
Device#2	3.7	0.32		8.94×10^{-9}
Device#3	5.0	2.2		3.54×10^{-7}
Device#4	6.1	2.8		4.07×10^{-7}
Device#5	4.6	7.0		5.87×10^{-7}
Device#6	4.0	0.41		1.02×10^{-8}
Device#7	3.8	0.23		7.42×10^{-9}
Device#8	4.7	0.17		8.34×10^{-9}

Supplementary Note 5: Overview of the BPVE in various materials.

We conducted a comprehensive comparison of the BPVE in Te with that observed in various other materials, assessing both the photoresponse spectrum and short-circuit photocurrent density (j_{sc}). The maximum response wavelength for materials exhibiting the BPVE is detailed in Supplementary Table 3. Notably, our analysis reveals that the majority of investigated semiconductors exhibit responses limited to ultraviolet (UV) and visible light. To date, the occurrence of semiconductors with the infrared BPVE is rare, with organic tetrathiafulvalene-p-chloranil (TTF-CA) being an exception. However, the long-term instability and low mobility of organic semiconductors severely restrict their further applications. Excitingly, the stable Te displays an infrared BPVE with a response wavelength extending up to 3.8 μm in the mid-infrared spectrum, significantly broadening the range of BPVE in semiconductors.

The j_{sc} holds significant merit for both materials exhibiting the BPVE and broad-spectrum neuromodulation since the photogenerated current must be substantial enough to activate nerve cells. Supplementary Table 3 provides a detailed illustration of j_{sc} for different wavelengths in reported materials as a function of power density. Remarkably, the j_{sc} in Te with the BPVE under 1.31 μm light illumination reaches an impressive 70.4 A cm^{-2}. This value is orders of magnitude greater than that in other pure materials for

the ultraviolet and visible bands as well as devices optimized by vertical geometry[9,13], varied channel length[9], Schottky barrier[14], AFM tip[15], and buffer layer[16]. Semimetals exhibiting the BPVE demonstrate a similar photocurrent density, albeit requiring a much higher power density. Considering both the response wavelength and photocurrent value, Te with the infrared BPVE emerges as an excellent candidate for broad-spectrum neuromodulation.

Supplementary Table 3 | Comparison of maximum response wavelength and photocurrent density among previous materials with the BPVE.

Materials	Maximum of response wavelength (μm)	photocurrent density @ light wavelength	Power ($W\ cm^{-2}$)	Ref.
**Te**	**3.8**	**70.4 A cm^{-2} @1.31 μm**	**15.7**	**This work**
		**11.2 A cm^{-2} @1.31 μm**	**1.5**	
		**8.6 A cm^{-2} @1.31 μm**	**1**	
		**3.9 A cm^{-2} @1.31 μm**	**0.5**	
		**2.5 A cm^{-2} @1.31 μm**	**0.29**	
		**1.16 A cm^{-2} @1.31 μm**	**0.15**	
		**1.04 A cm^{-2} @1.31 μm**	**0.1**	
		**0.7 A cm^{-2} @1.31 μm**	**0.08**	
		**0.26 A cm^{-2} @1.31 μm**	**0.032**	
		**0.22 A cm^{-2} @1.31 μm**	**0.025**	
		**0.027 A cm^{-2} @1.31 μm**	**0.004**	
MoS_2	0.97	0.1 A cm^{-2} @638 nm	0.5	17,18
3R-MoS_2	0.77	10 A cm^{-2} @630 nm	1450	19
WS_2	0.92	0.2 A cm^{-2} @638 nm	1	10,20
$TaIrTe_4$	-	5 A cm^{-2} @4 μm	800	21
TaAs	-	656 A cm^{-2} @10.6 μm	20000	22
BP/WSe_2	0.77	25 A cm^{-2} @532 nm	100	23
TTF-CA	1.8	1×10^{-6} A cm^{-2} @Visible	0.1	24
PZTO	0.49	0.0019 cm^{-2} @UV	0.15	16
		0.005 cm^{-2} @UV	0.1	25
		1×10^{-8} A cm^{-2} @UV	0.032	26
OMPH	0.75	5×10^{-8} A cm^{-2} @450 nm	0.08	27
MPI	0.87	1×10^{-4} A cm^{-2} @Visible	0.025	13,28
		0.02 A cm^{-2} @Visible	0.1	
KBNNO	0.88	1×10^{-7} A cm^{-2} @UV	0.004	29
BFO	0.78	1.02×10^{-4} A cm^{-2} @405 nm	0.29	30
		10.2 A cm^{-2} @405 nm	40	15
BFCO	0.97	0.001 A cm^{-2} @635 nm	1.5	31
BTO	0.45	1×10^{-8} A cm^{-2} @360 nm	0.5	26

		7×10^{-6} A cm^{-2} @360 nm	0.5	9

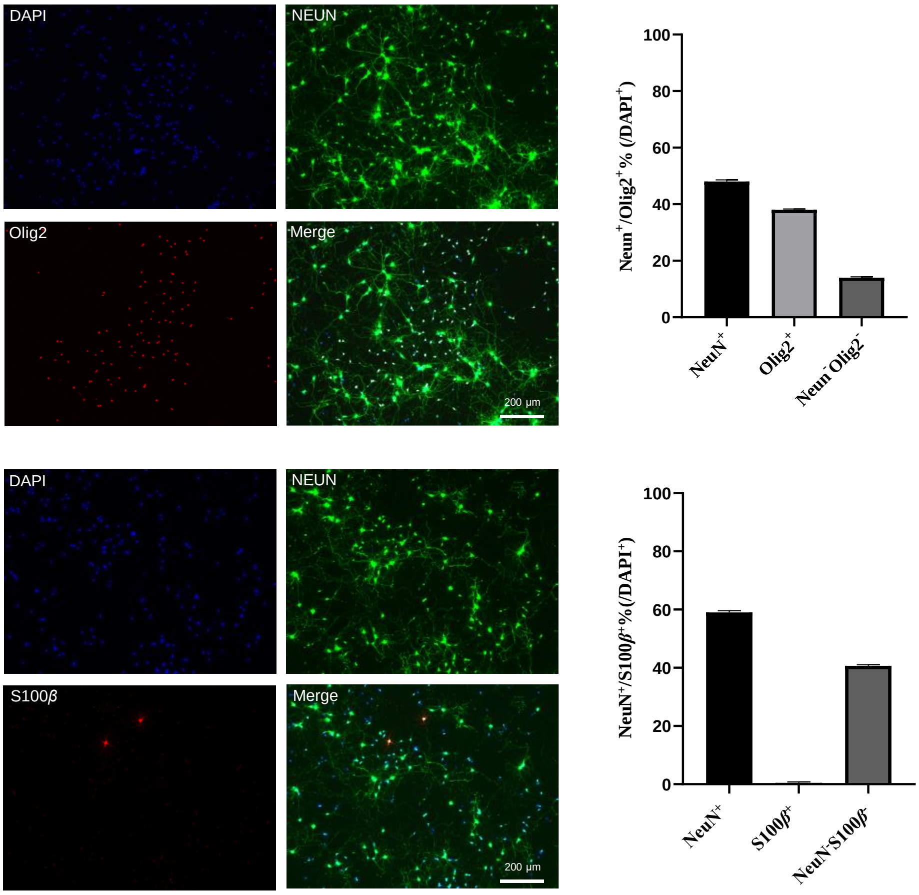

Supplementary Fig. 18 | Proportion of oligodendrocytes, astrocytes, and neurons. **a,** Confocal microscopy images of DIV 9 mouse primary cortex neurons. The oligodendrocytes and neurons were labeled by olig2 (red) and NEUN (green), respectively. **b,** Statistical histogram of neuronal and oligodendrocytes proportions. **c,** confocal microscopy images of DIV 9 neurons. S100*β* and NEUN were used to mark the astrocytes and neurons, respectively. **d,** Statistical histogram of neuronal and astrocyte proportions. Data are presented as mean and SEM.

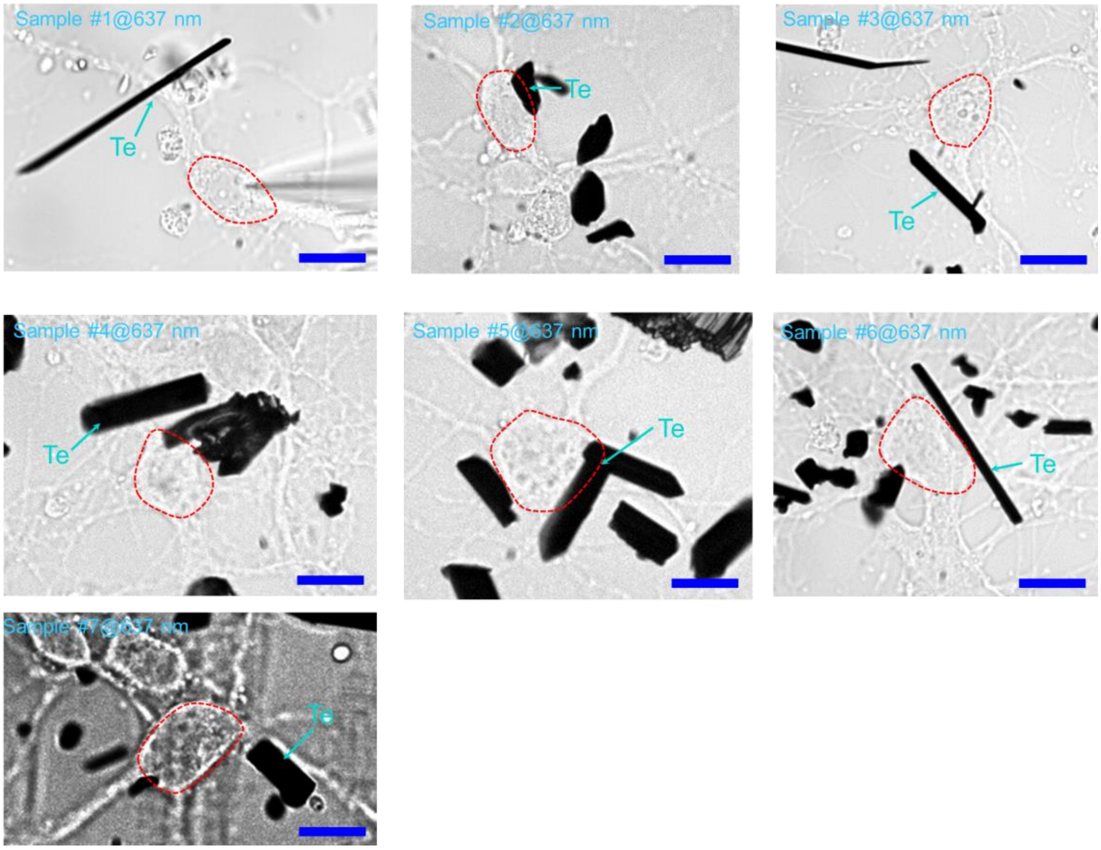

Supplementary Fig. 19 | Summary of microscopy images of the recorded neurons that are in contact with Te nanoflakes in response to 637 nm light. The somata of neurons and Te nanoflakes are marked by dashed red lines and cyan arrows, respectively. Te nanoflakes were founded on the somata of neurons (Sample #2, #5, #6). Te nanoflakes were observed on the dendrites of neurons (Sample #1, #3, #4, #7). Scale bars represent 10 μm.

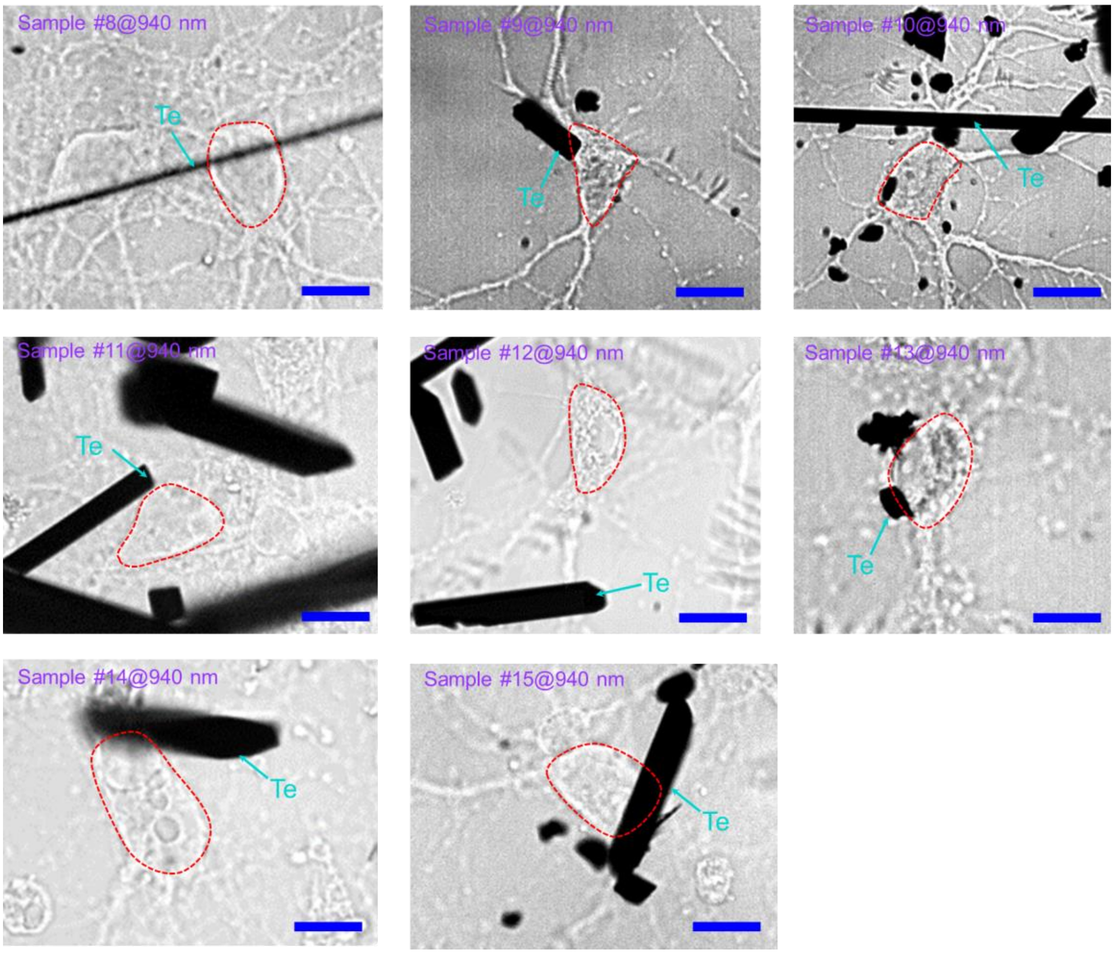

Supplementary Fig. 20 | Summary of microscopy images of the recorded neurons with Te nanoflakes for 940 nm light. The somata of neurons and Te nanoflakes are marked by dashed red lines and cyan arrows, respectively. Te nanoflakes were founded on the somata of neurons from Samples #8, #13, #14, and #15. Four Te nanoflakes were visualized on the dendrites of neurons from Samples #9, #10, #11, and #12. Scale bars represent 10 µm.

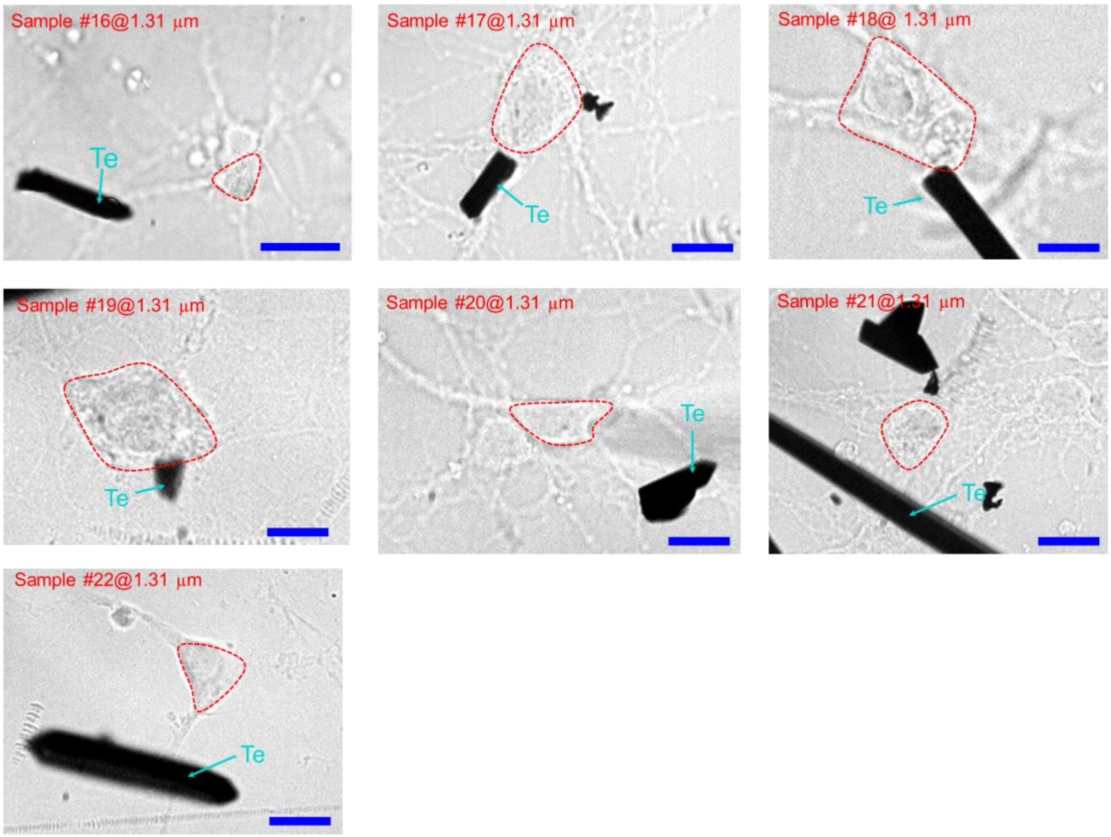

Supplementary Fig. 21 | Summary of microscopy images of the recorded neurons with Te nanoflakes for 1.31 μm light. The somata of neurons and Te nanoflakes are marked by dashed red lines and cyan arrows, respectively. Te nanoflakes were founded on the somata of neurons (Sample #17, #18, #19) and the dendrites of neurons (Sample #20, #21, #22). Scale bars represent 10 μm.

Supplementary Note 6: Degradation of Te.

Te were immersed in an artificial cerebrospinal fluid (ACSF) solution. The concentration of Te is 5 μg/mL. We obtained images of Te in the ACSF solution over different time periods, as shown in Supplementary Fig. 22a. After 21 days, the sizes of Te nanoflakes remain essentially unchanged (Supplementary Figs. 22b-c). To further reveal the ACSF solution on Te nanoflakes, we performed transmission electron microscopy (TEM), selected-area electron diffraction (SAED), and energy-dispersive X-ray spectroscopy (EDS) characterizations on Te nanoflakes with 21 days. Supplementary Figure 23a shows a low-magnification TEM image of the Te. EDS mapping measurement displayed in Supplementary Fig. 23b shows the ions from the

ACSF solution absorbed on the surface of Te nanoflake. Sharp diffraction in the SAED image (Supplementary Fig. 23c) still exhibits the single-crystalline of Te nanoflake after 21 days. Furthermore, the high-magnification TEM image in Supplementary Fig. 23d reveals an interplanar spacing distance of 0.59 nm, corresponding to the (0001) lattice planes of Te. These findings indicate that Te do not degrade in the ACSF solution even after 21 days, which is promising for long-term neural regulation.

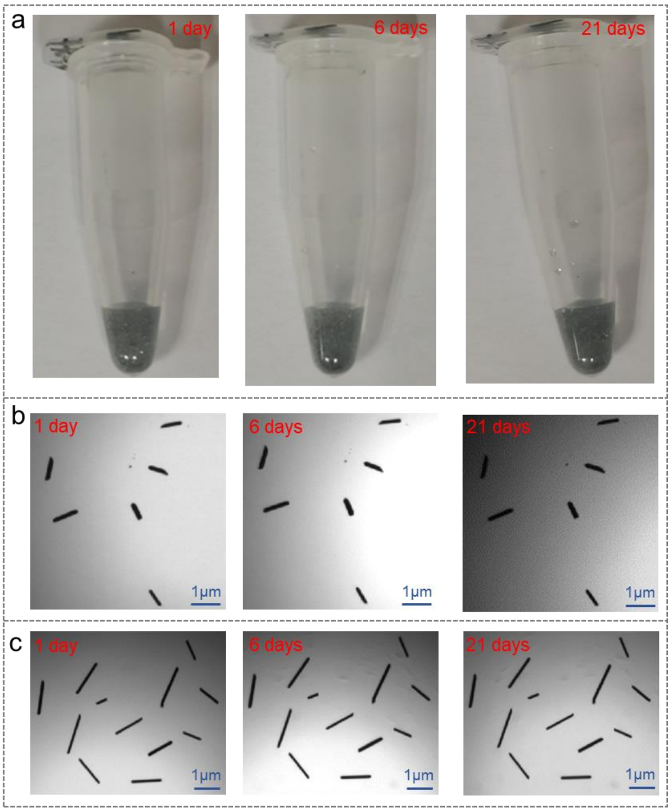

Supplementary Fig. 22 | Te in ACSF solution during different days. a, Images of Te nanoflakes in ACSF solution during different days. **b-c,** Optical microscope images of Te nanoflakes in ACSF solution during different days.

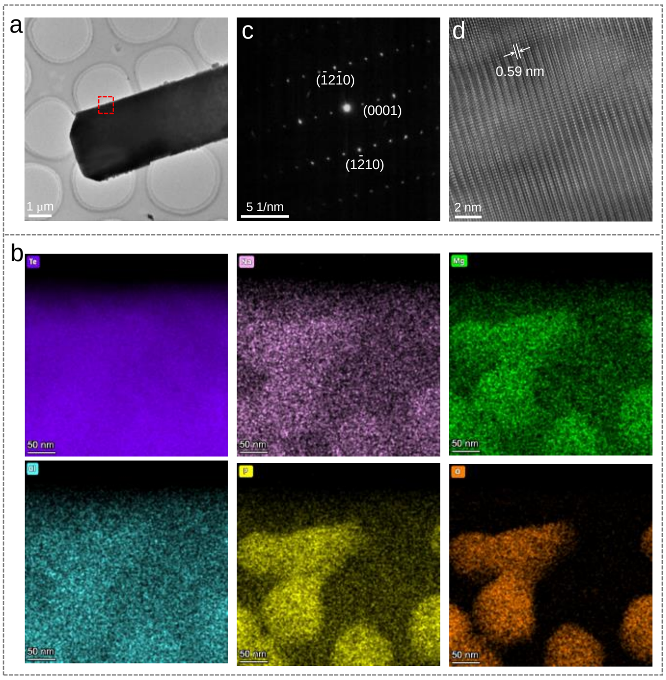

Supplementary Fig. 23 | Characterizations of Te nanoflakes in ACSF solution. a, Low-magnification TEM image of the Te nanoflakes in ACSF solution after 21 days. **b,** EDS mapping images of Te in the ACSF solution after 21 days. **c-d,** SAED, and high-magnification TEM images of the Te nanoflakes in ACSF solution after 21 days.

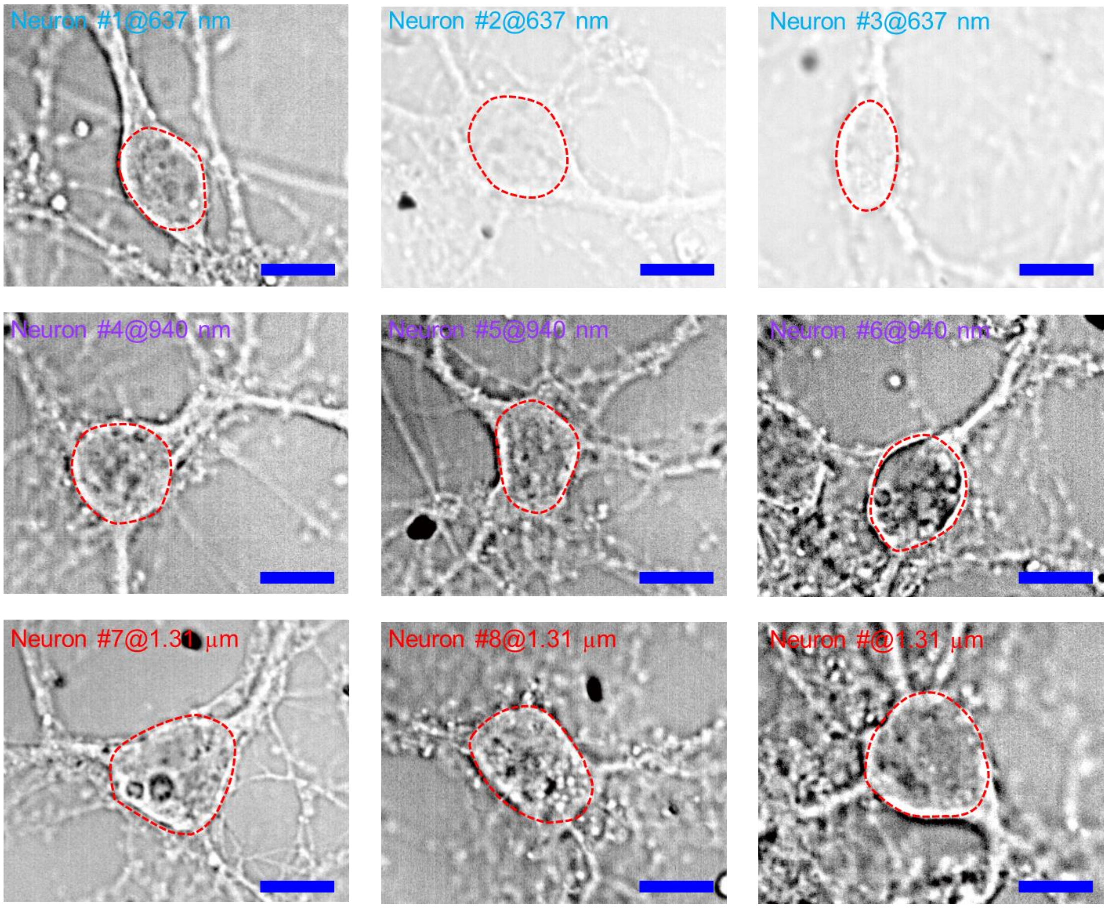

Supplementary Fig. 24 | Summary of microscopy images of the recorded neurons without Te. Neurons were cultured in the medium containing no Te nanoflakes. The somata of neurons are marked by dashed red lines. Scale bars represent 5 μm.

Supplementary Note 7: ACS solution pH and condition of neurons after long illumination cycles.

For the measurement of solution pH, we prepared freshly made ACSF solution without 95% O_2 and 5% CO_2. Once the pH of the ACSF solution stabilized, we mixed the ACSF solution with Te nanoflakes and added it to a 24-well plate, ensuring that each well contained 2.5 ml of the ACSF solution. As a control, we included wells with ACSF and Te nanoflakes without illumination. We measured the pH values of the ACSF solution every 5 minutes under different conditions. The results are shown in Figure R4.3. Comparing the pH of the ACSF solution without Te nanoflakes to those with Te nanoflakes after 1310 nm illumination revealed no significant differences. This indicates that Te nanoflakes do not alter the pH of ACSF solutions under long

illumination cycles. Additionally, there was no significant difference in pH between the two groups of ACSF with Te nanoflakes, regardless of whether they were illuminated at 1310 nm or not.

Primary cortical neurons were obtained from embryonic 18-day ICR mice and cultured for 9 days *in vitro*. Te nanoflakes were then added to the culture medium. After 48 hours of co-culture, neurons were irradiated with laser light at a wavelength of 1.31 μm for 10, 15, and 20 minutes, respectively. Immunohistochemical staining was performed, and optical microscope images of neurons and Te nanoflakes with different illumination times are presented in Supplementary Fig. 27. Cleaved caspase-3 staining was not observed in neurons after 10, 15, and 20 minutes of light irradiation, indicating that long-term illumination does not induce apoptosis, demonstrating that prolonged stimulation of neurons does not lead to cell death.

Electrophysiological experiments were further conducted to assess changes in the resting membrane potential (RMP) after long-term illumination. Whole-cell patch-clamp recordings of cultured cortical neurons at DIV11 were performed. A series of action potentials were generated following illumination with different wavelengths (637 nm, 940 nm, and 1.31 μm). We measured the RMP before and after 5 minutes of illumination. The results, shown in Supplementary Fig. 28, indicate no significant differences in the RMP of neurons before and after 5 minutes of continuous illumination.

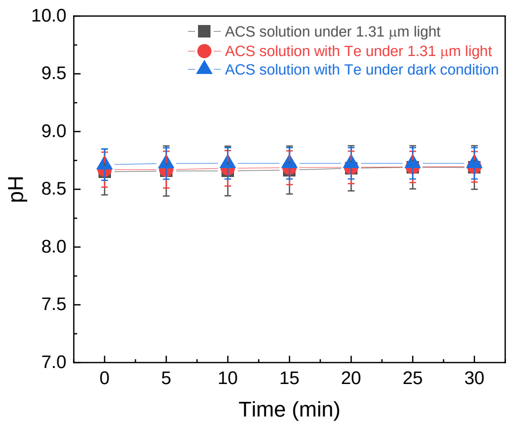

Supplementary Fig. 25 | The pH changes of the ACSF solution under 1.31 μm light illumination were recorded over time. After being co-cultured with Te nanoflakes for 2 days at DIV9, the neurons' culture medium was replaced with ACSF solution. The pH

of the ACSF solution was measured at 0 minutes and at 5-minute intervals thereafter. The frequency and duration of 1.31 μm light are 40 Hz and 4 ms, respectively. The power density used is 98.1 mW mm^{-2}. The results are obtained from six sets of experiments for every condition.

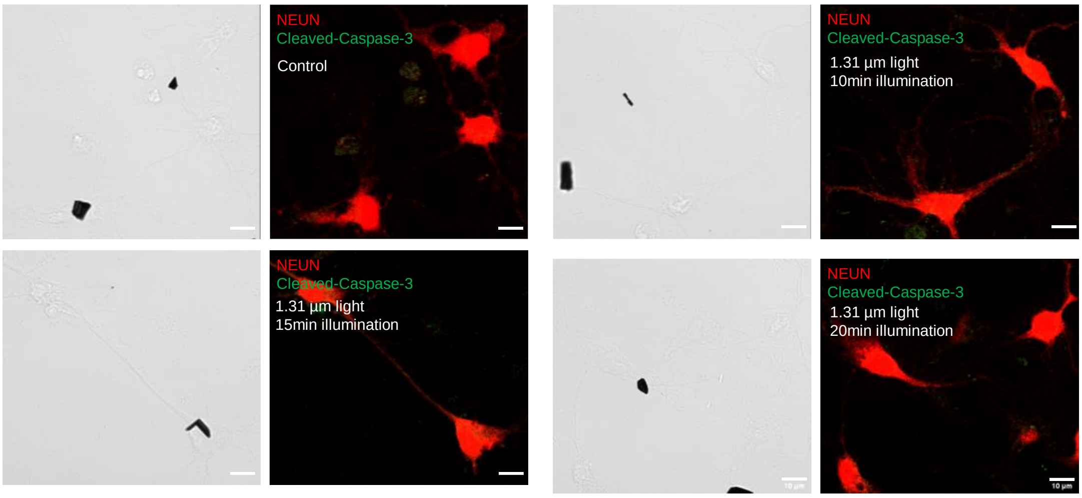

Supplementary Fig. 26 | No apoptosis was observed in neurons co-cultured with Te nanoflakes under 1.31 μm light illumination at various time intervals. The frequency and duration of 1.31 μm light are 40 Hz and 4 ms, respectively. The power density used is 98.1 mW mm^{-2}.

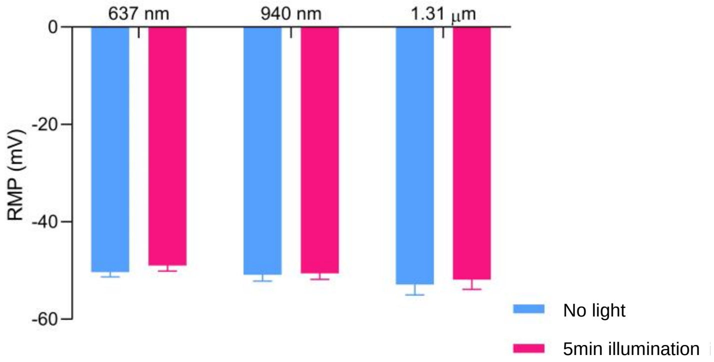

Supplementary Fig. 27 | Resting membrane potential of neurons was measured under 637 nm, 940 nm, and 1.31 μm light illumination for 5 minutes. The frequency and duration of 1.31 μm light are 40 Hz and 4 ms, respectively. The power density used is 98.1 mW mm^{-2}.

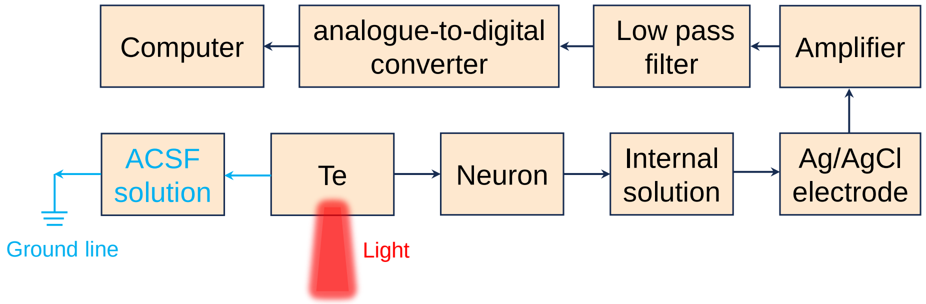

Supplementary Fig. 28 | Photocurrent signal diagram of Te-based patch-clamp recording.

Supplementary Note 8: Diameters and power densities of light with different wavelengths.

In our study, we used a power meter to obtain the power of different-wavelength light. The duration of 637 nm, 940 nm, and 1.31 μm light is 10 ms. The maximum powers of 637 nm, 940 nm, and 1.31 μm light are 8.18 μW, 1.75 μW, and 1.65 μW, respectively. Furthermore, we conducted spot analyzer measurements and determined the diameters of the light spots to be 19.8 μm, 45.2 μm, and 74.6 μm for 637 nm, 940 nm, and 1.3 μm light, respectively, as shown in Supplementary Fig. 29. As a result, the power densities of 637 nm, 940 nm, and 1.3 μm light are 26.57 mW mm^{-2}, 1.09 mW mm^{-2}, and 0.38 mW mm^{-2}, respectively. The maximum power densities of 637nm, 940 nm, and 1.3 μm light in our experiment are smaller than those recommended by the International Commission on Non-Ionizing Radiation Protection (ICNIRP).

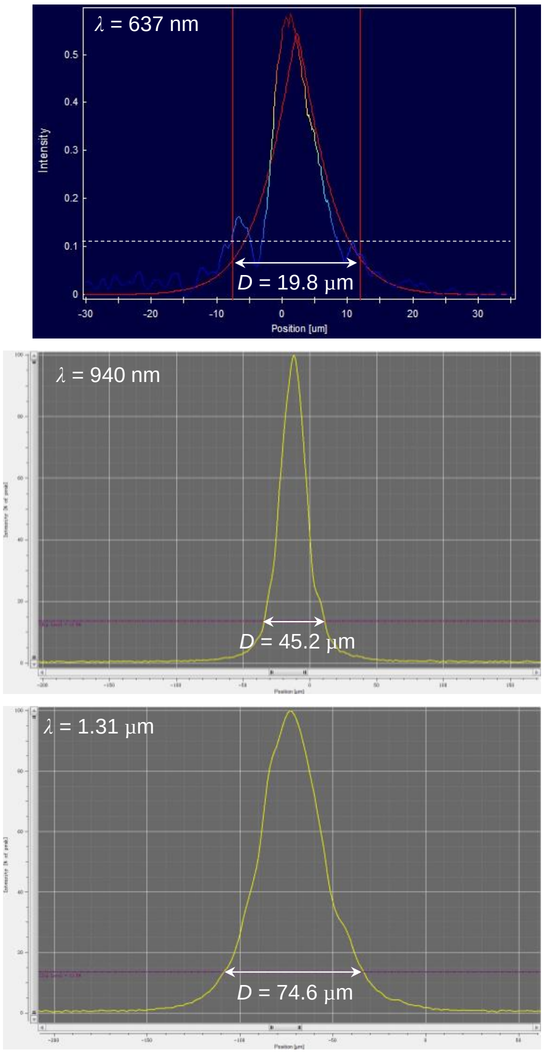

Supplementary Fig. 29 | Spot size of light with different wavelengths. Diameter distribution of 637 nm, 940 nm, and 1.3 μm light obtained by the Thorlabs Beam Profilers.

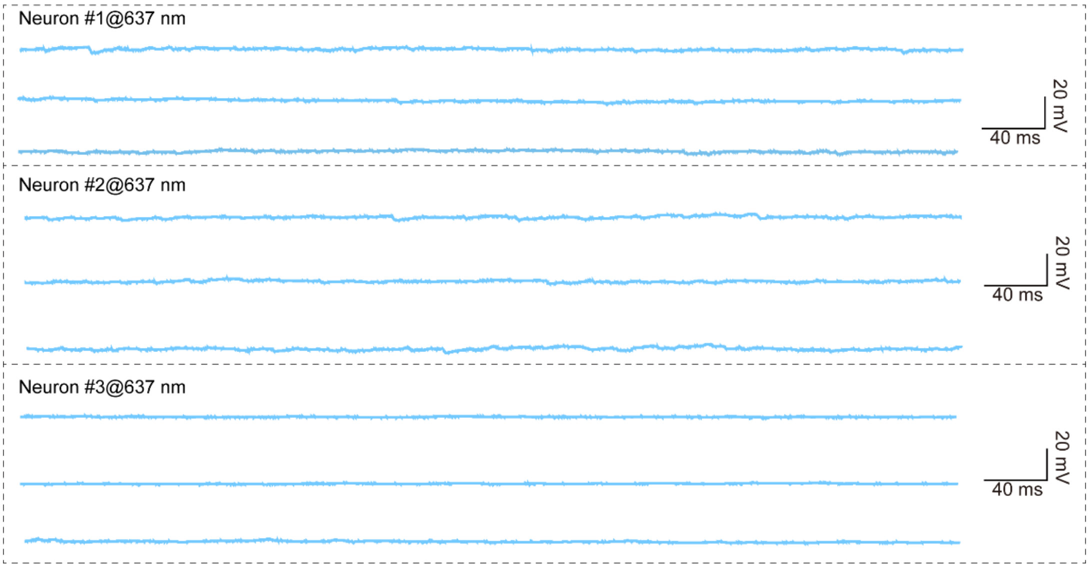

Supplementary Fig. 30 | Action potentials are not generated in the neurons without Te under 637 nm light illumination. 637 nm light was used to illuminate neuron #1, neuron #2, and neuron #3 as the power increased from 5.5 × 10^3 mW mm^{-2} to 5.3 × 10^4 mW mm^{-2} then to 2.6 × 10^5 mW mm^{-2}.

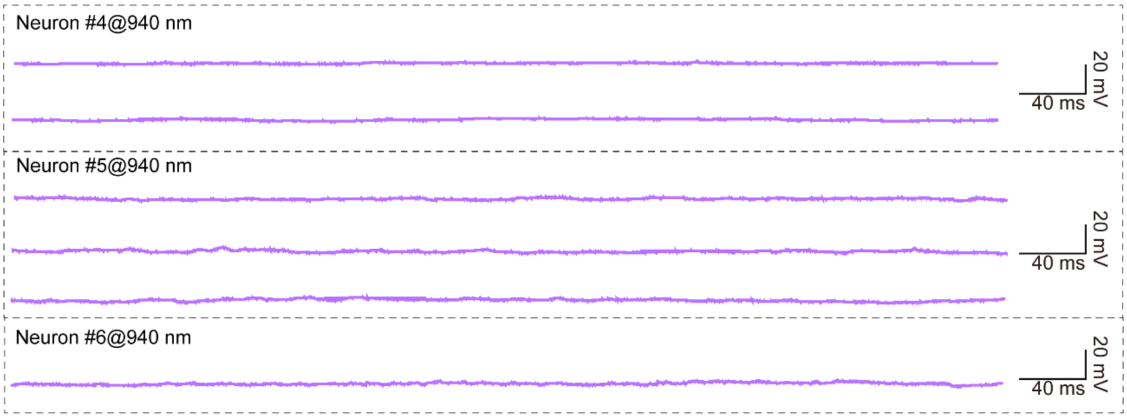

Supplementary Fig. 31 | Action potentials are not generated in the neurons without Te under 940 nm light illumination. 940 nm light with different power was used to illuminate neuron #4 (3.9 × 10^3 mW mm^{-2} and 6.8 × 10^3 mW mm^{-2}), neuron #5 (1.9 × 10^3 mW mm^{-2}, 3.8 × 10^3 mW mm^{-2}, and 6.8 × 10^3 mW mm^{-2}), and neuron #6 (1.9 × 10^3 mW mm^{-2}).

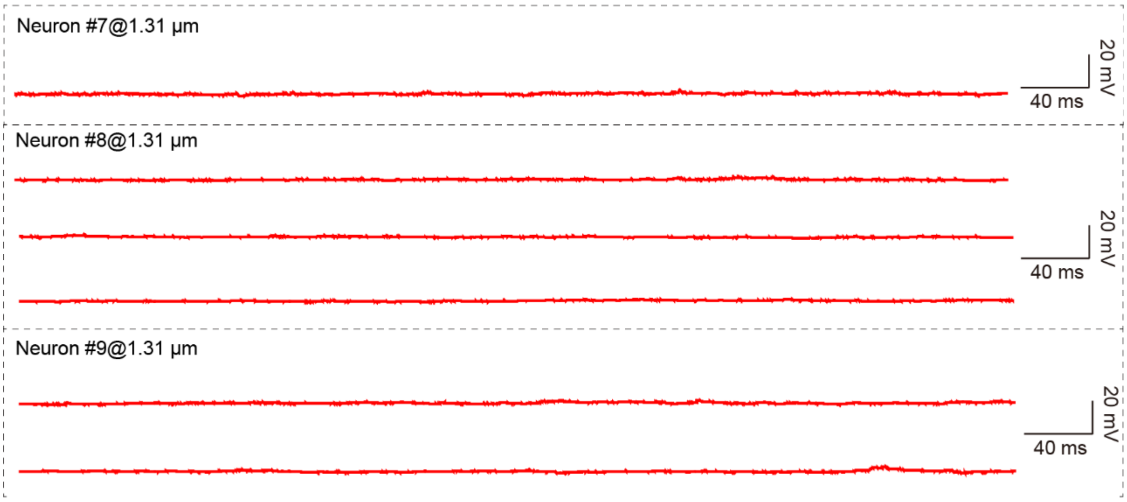

Supplementary Fig. 32 | Action potentials are not evoked in the neurons without Te under 1.31 μm light illumination. 1.31 μm light with different power was applied to illuminate neuron #7 (3.3×10^3 mW mm^{-2}), neuron #8 (1.1×10^3 mW mm^{-2}, 2.1×10^3 mW mm^{-2}, and 2.3×10^3 mW mm^{-2}), and neuron #9 (2.3×10^3 mW mm^{-2} and 3.3×10^3 mW mm^{-2}).

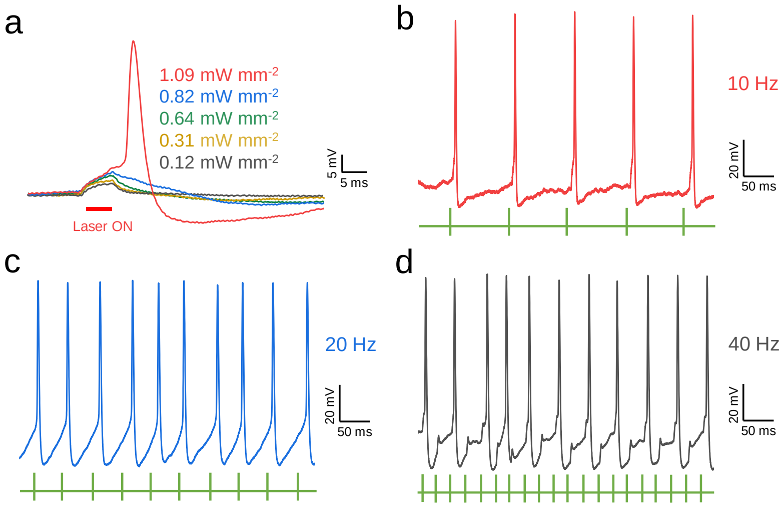

Supplementary Fig. 33 | Neuromodulation of mouse primary cortical neurons with Te under 940 nm light irradiation. a, Electrophysiology current-clamp traces of membrane voltage in the primary cortical neurons co-cultured with Te nanoflakes stimulated by 940 nm light with different energy densities. The frequency and duration are 10 Hz and 5 ms, respectively. **b-d,** Electrophysiology current-clamp traces of membrane voltage in the primary cortical neurons co-cultured with Te nanoflakes

stimulated by 940 nm light with different frequencies. The duration and power density are 5 ms and 1.09 mW mm^{-2}, respectively.

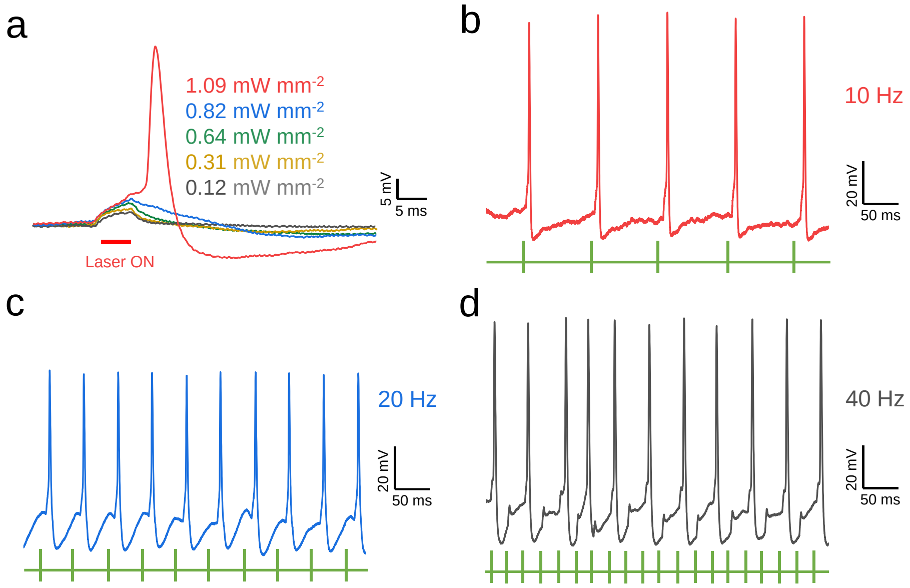

Supplementary Fig. 34 | Neuromodulation of mouse primary cortical neurons with Te under 637 nm light irradiation. a, Electrophysiology current-clamp traces of membrane voltage in the primary cortical neurons co-cultured with Te nanoflakes stimulated by 637 nm light with different energy densities. The frequency and duration are 10 Hz and 5 ms, respectively. **b-d,** Electrophysiology current-clamp traces of membrane voltage in the primary cortical neurons co-cultured with Te nanoflakes stimulated by 637 nm light with different frequencies. The duration and power density are 5 ms and 25.2 mW mm^{-2}, respectively.

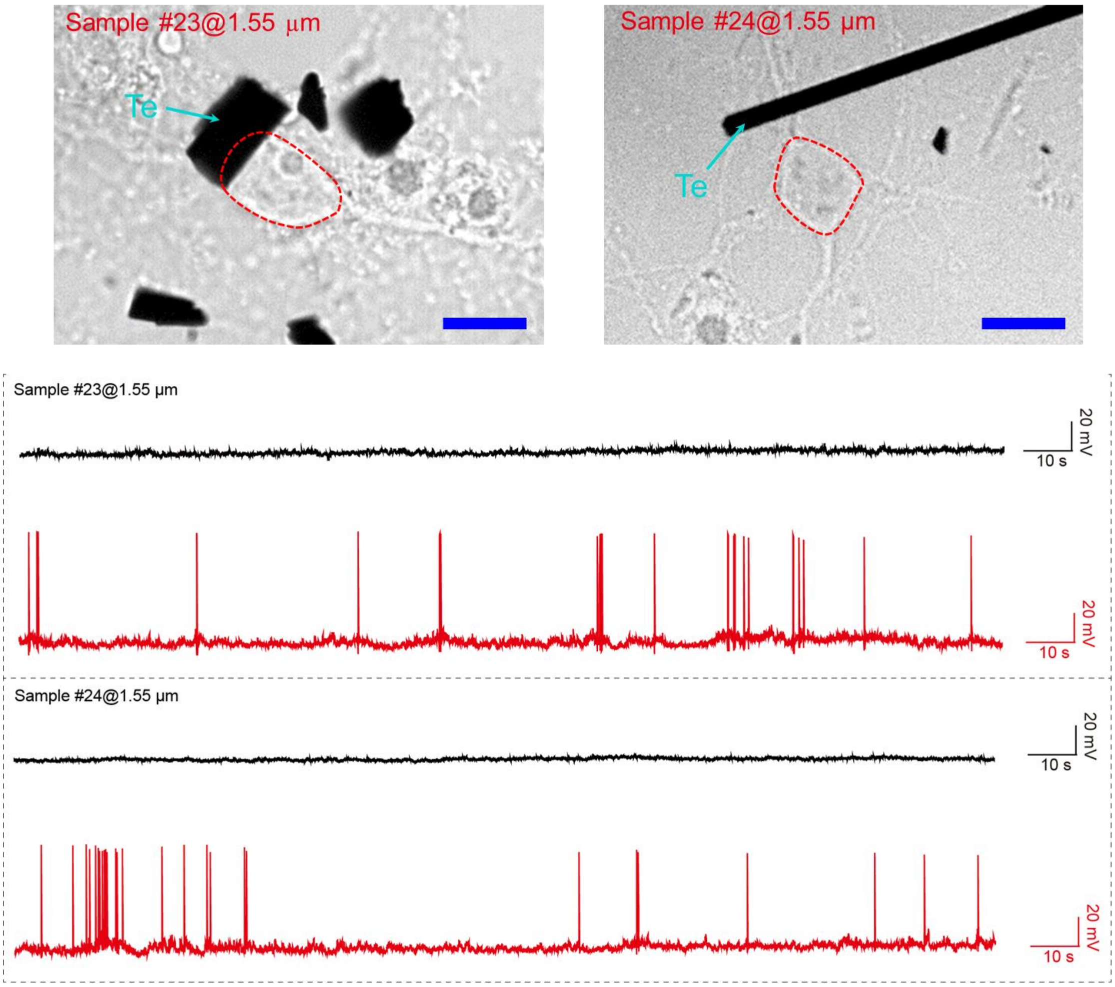

Supplementary Fig. 35 | Action potentials are elicited in the neurons with Te under 1.55 µm light irradiation. Neurons that are in contact with Te were recorded under no light (black lines), followed by 1.55 µm light illumination (blue lines). Note that many action potentials were elicited by 1.55 µm light. The power density of 1.55 µm light is 1.7×10^2 mW mm^{-2} and 2.2×10^2 mW mm^{-2}, respectively. Te nanoflakes were observed on the soma of neuro (Sample #23) and the dendrite of neuron (Sample #24), respectively. Scale bars represent 10 µm.

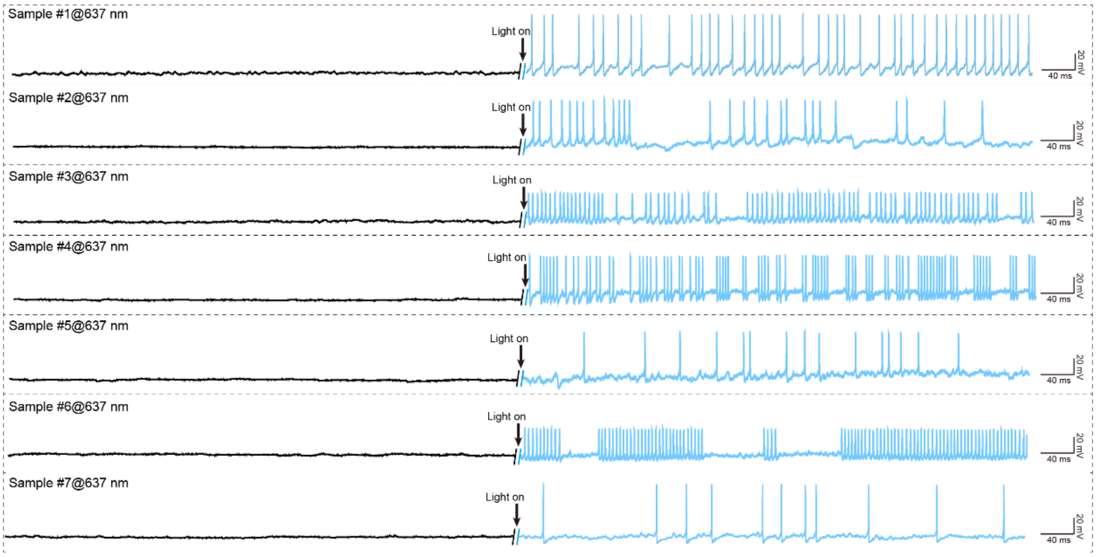

Supplementary Fig. 36 |Action potentials are evoked in the neurons with Te under 637 nm light irradiation. Neurons that are in contact with Te were recorded under no light (black lines), followed by 637 nm light illumination (blue lines). Note that a series of action potentials were elicited by 637 nm light. Light exposure applied to neurons is continuous and lasts for 6 seconds. Power densities are 104.3, 111.6, 121.3, 90.7, 94.5, 98.6, and 106.7 mW mm^{-2}.

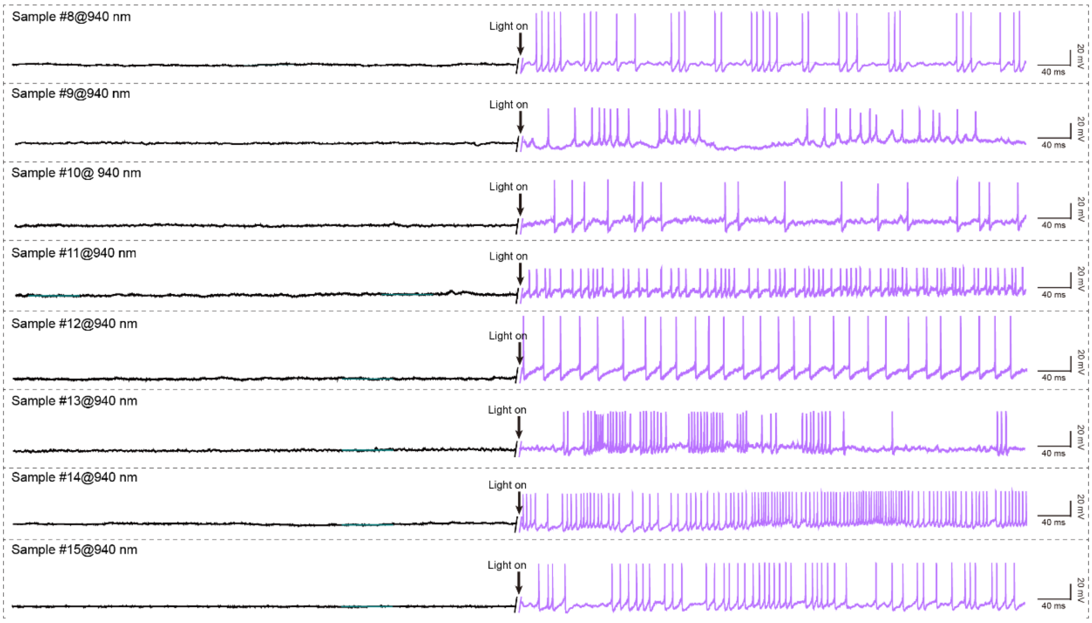

Supplementary Fig. 37 | Action potentials are elicited in the neurons with Te under 940 nm light irradiation. Neurons that are in contact with Te were recorded under no light (black lines), followed by 940 nm light illumination (purple lines). Note that a

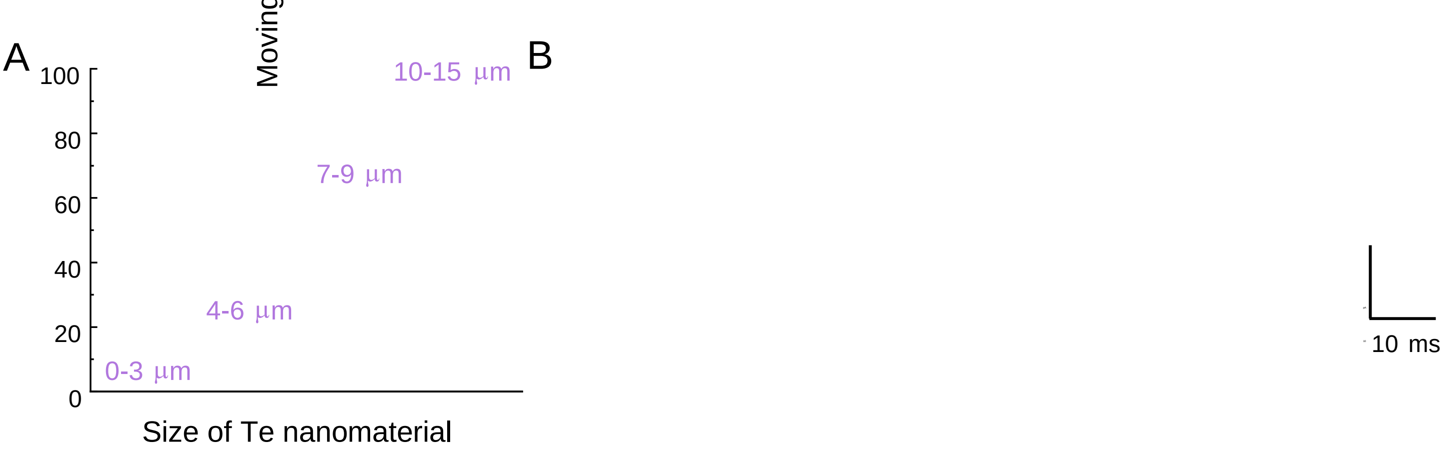

is continuous and lasts for 6 seconds. Power densities are 15.5, 13.4, 9.82, 6.84, 11.4, and 7.32 mW mm^{-2}.

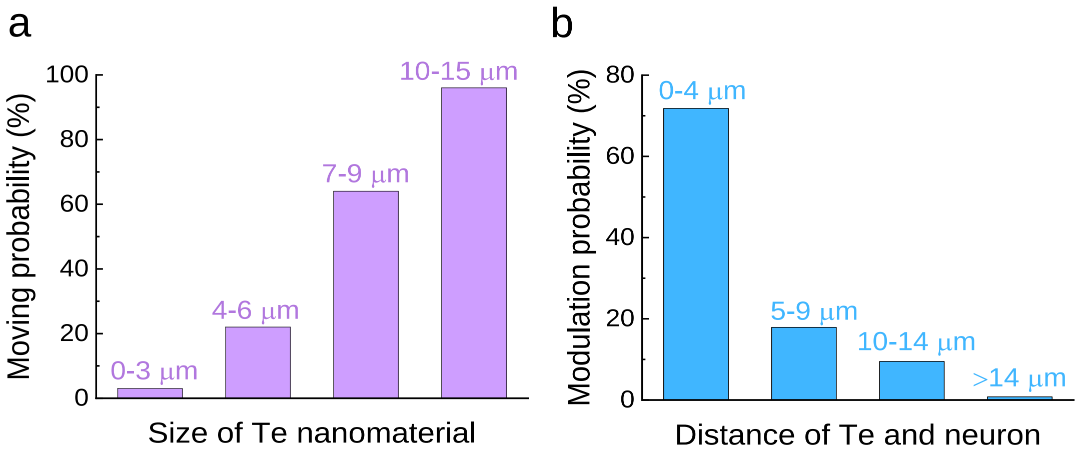

Supplementary Fig. 39 | Factors of Te-based neuromodulation. a, Moving probability of Te nanoflakes away from neurons as the size of Te nanoflakes increases. To simulate the scene of a slight movement for neurons and Te nanoflakes, we put the petri dish containing Te nanoflakes and neurons on the orbital shaker. The shaker was

set to a speed of approximately 120 rpm, and the duration of shaking was set to 5 minutes. We conducted this experiment with 200 groups of neurons and Te nanoflakes. **b**, Neuromodulation probability as the relationship of the distance of Te nanoflakes and soma of neurons under 1.31 μm light illumination.

Supplementary Note 9: Photocurrent and photovoltage generated by the Te nanoflakes using the patch clamp setup.

We employed a patch-clamp configuration to assess the photocurrents of a Te nanoflake responding to light of 637 nm, 940 nm, and 1.31 μm wavelengths in neuromodulation, each lasting 10 ms. The Te nanoflake, situated at the tip of a recording pipette, was directly illuminated by the light, and the resulting currents were recorded in the voltage-clamp mode at a holding potential of zero millivolts. The photocurrents induced by light illumination were sustained and non-capacitive, with their amplitudes being directly proportional to the power of the incident light and capable of exceeding 20 pA (Supplementary Fig. 41). We further measured the photocurrent changes after multi-cyclic pulses. As the number of cyclic pulses of 1.31 μm light increases up to 1000, the photocurrents generated in the Te nanoflakes rapidly decrease. Beyond 1000 pulses, the photocurrents reach a plateau and no further changes are observed up to 2×10^5 pulses (Supplementary Fig. 42). Additionally, we have measured the photovoltage generated by the Te nanoflakes using the patch clamp setup in current clamp. The power density of 0.11 mW mm^{-2} was sufficient to generate photocurrent. However, we did not observe any photovoltage in the Te nanoflakes. Even as the power density of the 1.31 μm light was gradually increased, no photovoltage was generated (Supplementary Fig. 43).

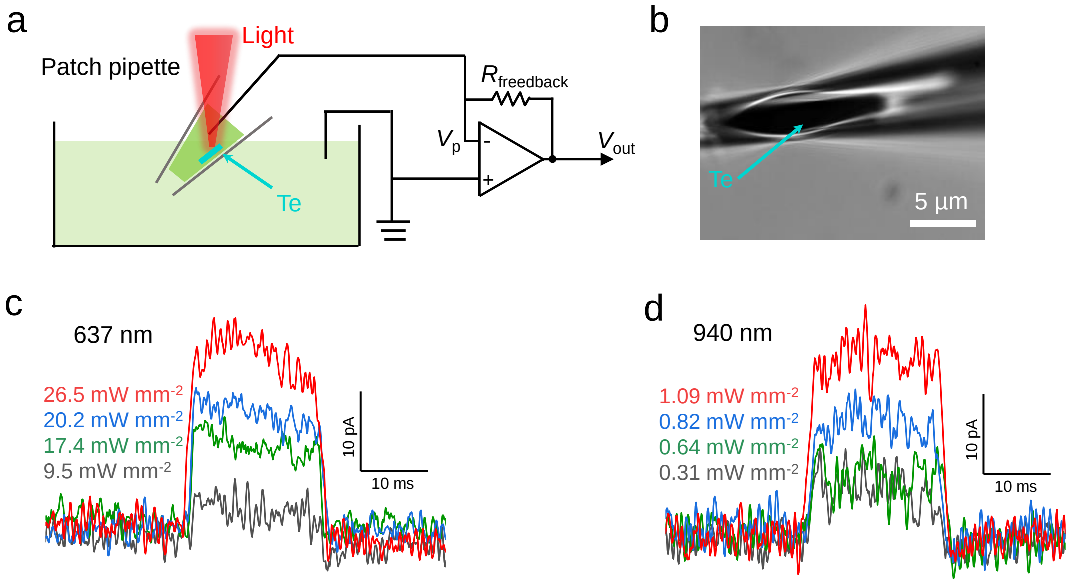

Supplementary Fig. 40 | Generated photocurrent of a Te nanoflake in optical neuromodulation. a, Schematic of photocurrent measurement setup for a Te nanoflake. V_p, $R_{feedback}$, and V_{out} represent the pipette voltage, the resistance of a feedback resistor, and the output voltage, respectively. **b,** Microscope image of a Te nanoflake in the pipette. **c-d,** Photocurrent traces from a Te nanoflake with the 637 nm and 940 nm. The frequency and duration of the light are 1 Hz and 10 ms, respectively.

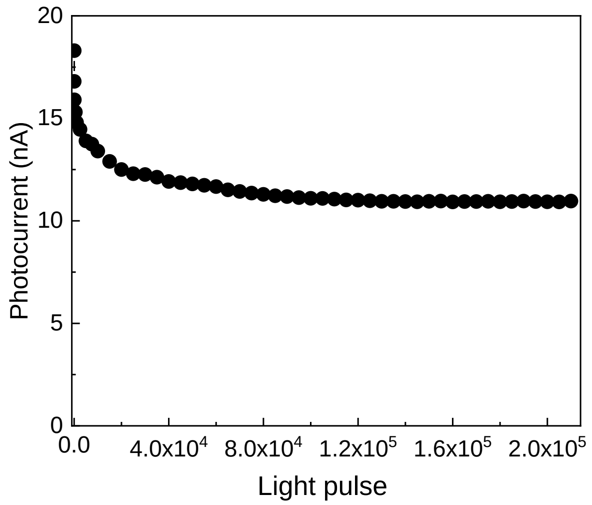

Supplementary Fig. 41 | Photocurrent of Te nanoflake related to 1.31 μm light pulses. The frequency and duration of 1.31 μm light are 40 Hz and 4 ms, respectively. The power density is 0.11 mW mm^{-2}.

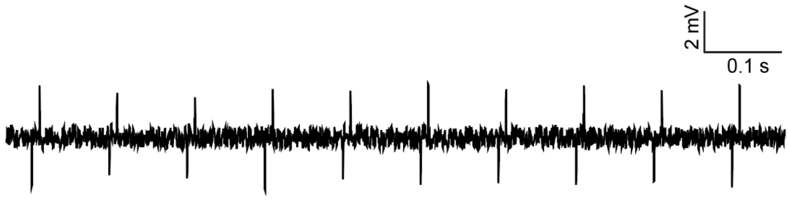

Supplementary Fig. 42 | Photovoltage generated by the Te nanoflake using the patch clamp setup in the current clamp. The red bars represent light pulses with a 10 ms duration.

Supplementary Note 10: Temperature changes of Te under light irradiation.

We performed temperature measurements. Te nanoflakes were grown on SiO_2/Si substrate. The area is approximately 8×7 mm^2 and fully covered by the light, as shown in Supplementary Fig. 43a. Supplementary Figures 43b-33c display the optical microscope image and Raman spectra of Te. Te on the SiO_2/Si substrate were illuminated under 60s light with different wavelengths. 0s and 60s thermal images were obtained by a Teledyne FLIR infrared camera, shown in Supplementary Figs. 43d-f. After comparison, a maximum 0.18 °C temperature change is observed. These results indicate that the BPVE of Te is dominant in optical neuromodulation.

We also conducted a simultaneous measurement of temperature and neuronal action potential produced by laser stimulation. Supplementary Figure 44 shows the schematic of the simultaneous temperature measurement setup of temperature and neuronal action potentials produced by laser stimulation. A thermometer pipette, which measures the change in temperature via an alteration in the pipette resistance, is placed within ~ 2 μm of the neuron/Te nanoflake interface. Simultaneously, the neuron is recorded by a patch pipette from the other side, shown in Supplementary Fig. 45a. The relationship between measured pipette resistances and corresponding Te surface temperatures is shown in Supplementary Fig. 45b. A change in the temperature of the Te surface following laser stimulation of the neuron-Te is documented based on the linear relationship between a change in the pipette resistance and a change in the temperature of the Te surface, which is plotted by a change in the pipette resistance corresponding to a gradual decrease in the bath temperature from 32.5.

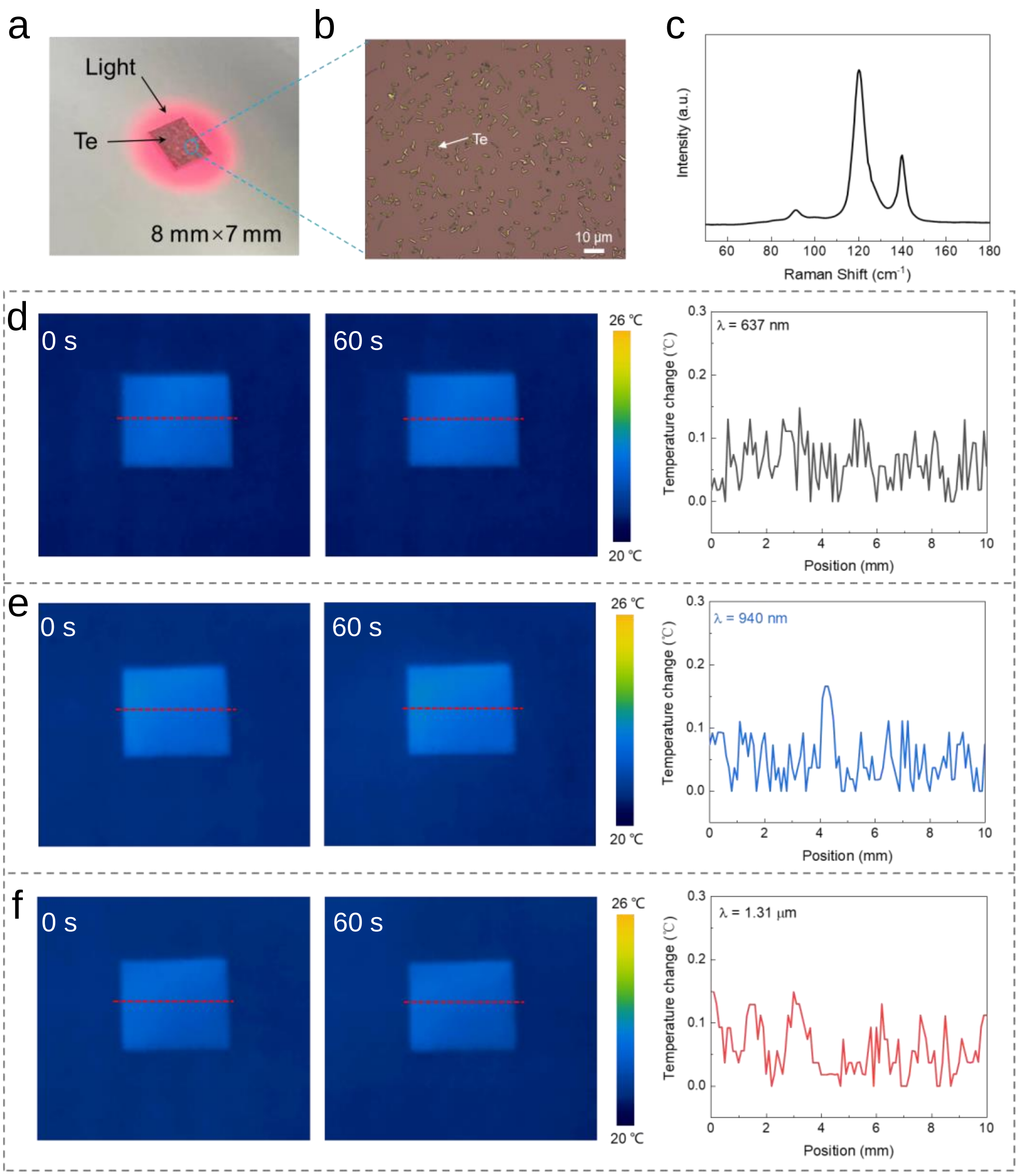

Supplementary Fig. 43 | Temperature changes of Te under light irradiation. a, Image of Te on Si substrate illuminated by the light, which is used to measure the temperature change. **b-c,** Microscope image and Raman spectra of Te. The golden yellow shapes represent Te. **b-c,** Thermal images and temperature changes of Te under 60s illumination by 637nm, 940 nm, and 1.31 μm light. The power densities of 637 nm, 940 nm, and 1.31 μm light are 2.6×10^4 mW mm^{-2}, 3.7×10^3 mW mm^{-2}, and 1.1 $\times10^3$ mW mm^{-2}, respectively. Temperature is extracted along the red lines in 0s and 60s thermal images. The temperature change profile is the difference between 0s and 60s lines.

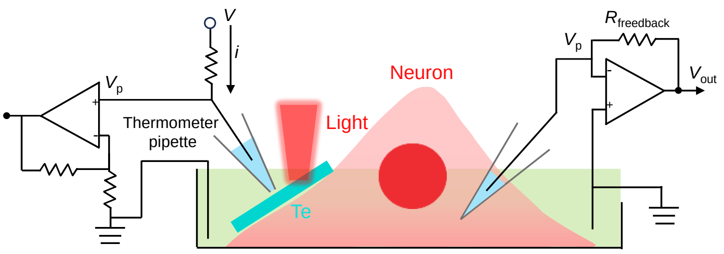

Supplementary Fig. 44 | Schematic of temperature measurement setup for simultaneous measurement of temperature and neuronal action potentials produced by laser stimulation.

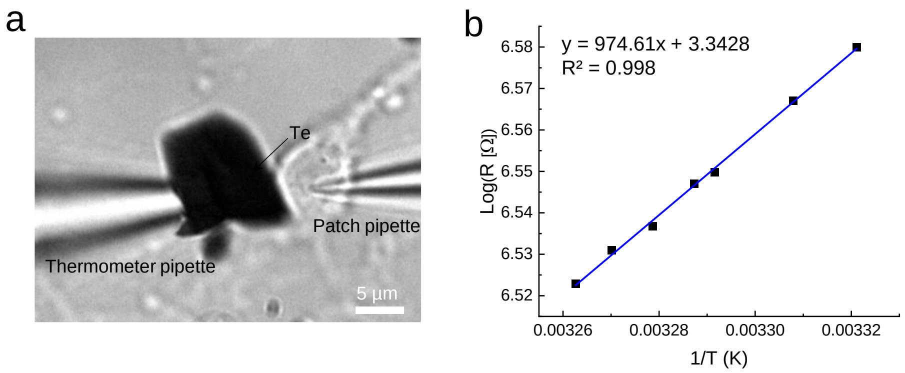

Supplementary Fig. 45 | Temperature measurement of the neuron and Te nanoflake. a, Microscopy image of a neuron during a simultaneous membrane voltage and temperature recording experiment. Thermometer pipette is placed within ~ 2 µm of the neuron/Te nanoflake interface. Neuron is current clamped by a patch pipette from the other side. **b,** The relationship between measured pipette resistances and corresponding bath temperatures. Calibration curves are fitted by a linear relationship.

Supplementary Table 4 | Comparison of photoelectric neuromodulation.

Photoelectric effect	Material structure	Maximum response wavelength	Stimulation wavelength (Power density)	Temperature increase (Irradiation time)	Heat	Ref.
**Bulk photovoltaic effect**	**A single Te**	**3.8 μm**	**637 nm (26.5 mW mm^{-2}), 940 nm (1.09 mW mm^{-2}), 1.31 μm (0.37 mW mm^{-2})**	**+0.18 ℃ (60 s)**	**No**	**This work**
P-n junction photovoltaic effect	P-type Si/ intrinsic Si/n-type Si nanowires	1.1 μm	532 nm (1.8×10^3 mW mm^{-2})	+0.36 ℃	No	32
	rr-P3HT/PCBM	670 nm	532 nm (10 mW mm^{-2})	/	No	33
Spectrum-selective upconversion	$NaYF_4$:Yb/Tm@ SiO_2 nanoparticles	/	980 nm (8.2×10^3 mW cm^{-2})	+0.3 ℃	No	34
	$NaYF_4$:Yb/Er nanoparticles	/	980 nm (5.3 mW mm^{-2})	/	No	35
	Lanthanide nanoparticles	/	980 nm (18.6 mW mm^{-2})	/	No	36
	NaYF4:Sc/Yb/Er nanoparticles	/	975 nm (4.1×10^4 mW mm^{-2})	/	No	37
	dye-sensitized core/shell nanoparticles	/	800 nm (2.3×10^3 mW mm^{-2})	/	No	38
Photothermal effect	pBBTV nanoparticles	/	1.06 μm (10 mW mm^{-2})	+16.7 ℃ (1.1 s)	Yes	39
	SPNsbc nanoparticles	980 nm	808 nm (1×10^5 mW mm^{-2})	+16.1 ℃ (38.6 s)	Yes	40
	Gold nanorods	920 nm	808 nm (8×10^3 mW mm^{-2})	+6 ℃ (120 s)	Yes	41
	Gold nanoparticles	/	532 nm (3.1×10^5 mW mm^{-2})	+9 ℃ (1 ms)	Yes	42
	Mesostructured silicon	/	532 nm (6.8×10^4 mW cm^{-2})	+5.8 ℃ (1.8 ms)	Yes	43
Photoacoustic effect	Polymer with polystyrene-block-poly (acryl acid)	/	1.03 μm (70 mW cm^{-2})	+8 ℃ (4 ns)	Yes	44
	Carbon nanotubes/silk scaffolds	/	1.03 μm (2.94 mW cm^{-2})	+0.7 ℃ (100 ms)	Yes	45

Reference

1 Ray, C. S., Fang, X. & Day, D. E. New Method for Determining the Nucleation and Crystal-Growth Rates in Glasses. *J. Am. Ceram. Soc.* **83**, 865-872, (2000).

2 Peng, M. *et al.* Blackbody-sensitive room-temperature infrared photodetectors based on low-dimensional tellurium grown by chemical vapor deposition. *Sci. Adv.* **7**, eabf7358, (2021).

3 Qiu, G. *et al.* Thermoelectric performance of 2D tellurium with accumulation contacts. *Nano*

Lett. **19**, 1955-1962, (2019).

4 Tutihasi, S., Roberts, G. G., Keezer, R. C. & Drews, R. E. Optical properties of tellurium in the fundamental absorption region. *Phys. Rev.* **177**, 1143-1150, (1969).

5 Amani, M. *et al.* Solution-Synthesized High-Mobility Tellurium Nanoflakes for Short-Wave Infrared Photodetectors. *ACS Nano* **12**, 7253-7263, (2018).

6 Akamatsu, T. *et al.* A van der Waals interface that creates in-plane polarization and a spontaneous photovoltaic effect. *Science* **372**, 68-72, (2021).

7 Guo, N. *et al.* Anomalous and highly efficient InAs nanowire phototransistors based on majority carrier transport at room temperature. *Adv. Mater.* **26**, 8203-8209, (2014).

8 Fang, H. & Hu, W. Photogating in low dimensional photodetectors. *Advanced Science* **4**, 1700323, (2017).

9 Zenkevich, A. *et al.* Giant bulk photovoltaic effect in thin ferroelectric $BaTiO_3$ films. *Phys. Rev. B* **90**, 161409, (2014).

10 Zhang, Y. J. *et al.* Enhanced intrinsic photovoltaic effect in tungsten disulfide nanotubes. *Nature* **570**, 349-353, (2019).

11 Wang, Q. *et al.* Ultrafast broadband photodetectors based on three-dimensional dirac semimetal Cd_3As_2. *Nano Lett.* **17**, 834-841, (2017).

12 Yuan, H. *et al.* Generation and electric control of spin–valley-coupled circular photogalvanic current in WSe_2. *Nat. Nanotechnol.* **9**, 851-857, (2014).

13 Xiao, Z. *et al.* Giant switchable photovoltaic effect in organometal trihalide perovskite devices. *Nat. Mater.* **14**, 193-198, (2015).

14 Ji, W., Yao, K. & Liang, Y. C. Bulk photovoltaic effect at visible wavelength in epitaxial ferroelectric $BiFeO_3$ thin films. *Adv. Mater.* **22**, 1763-1766, (2010).

15 Alexe, M. & Hesse, D. Tip-enhanced photovoltaic effects in bismuth ferrite. *Nat. Commun.* **2**, 256, (2011).

16 Cao, D. *et al.* High-Efficiency Ferroelectric-Film Solar Cells with an n-type Cu_2O Cathode Buffer Layer. *Nano Lett.* **12**, 2803-2809, (2012).

17 Moun, M., Singh, A., Tak, B. R. & Singh, R. Study of the photoresponse behavior of a high barrier Pd/MoS_2/Pd photodetector. *J. Phys. D: Appl. Phys.* **52**, 325102, (2019).

18 Jiang, J. *et al.* Flexo-photovoltaic effect in MoS_2. *Nat. Nanotechnol.* **16**, 894-901, (2021).

19 Dong, Y. *et al.* Giant bulk piezophotovoltaic effect in 3R-MoS_2. *Nat. Nanotechnol.* **18**, 36-41, (2023).

20 Ellmer, K. Preparation routes based on magnetron sputtering for tungsten disulfide (WS_2) films for thin-film solar cells. *Phys Status Solidi B* **245**, 1745-1760, (2008).

21 Ma, J. *et al.* Nonlinear photoresponse of type-II Weyl semimetals. *Nat. Mater.* **18**, 476-481, (2019).

22 Osterhoudt, G. B. *et al.* Colossal mid-infrared bulk photovoltaic effect in a type-I Weyl semimetal. *Nat. Mater.* **18**, 471-475, (2019).

23 Akamatsu, T. *et al.* A van der Waals interface that creates in-plane polarization and a spontaneous photovoltaic effect. *Science* **372**, 68-72, (2021).

24 Nakamura, M. *et al.* Shift current photovoltaic effect in a ferroelectric charge-transfer complex. *Nat. Commun.* **8**, 281, (2017).

25 Ichiki, M. *et al.* Photovoltaic effect of lead lanthanum zirconate titanate in a layered film structure design. *Appl. Phys. Lett.* **84**, 395-397, (2004).

26 Brody, P. S. High voltage photovoltaic effect in barium titanate and lead titanate-lead zirconate ceramics. *J. Solid State Chem.* **12**, 193-200, (1975).
27 Sun, Z. *et al.* A photoferroelectric perovskite-type organometallic halide with exceptional anisotropy of bulk photovoltaic effects. *Angew. Chem. Int. Edit.* **55**, 6545-6550, (2016).
28 Liu, Y. *et al.* Fast growth of thin $MAPbI_3$ crystal wafers on aqueous solution surface for efficient lateral-structure perovskite solar cells. *Adv. Funct. Mater.* **29**, 1807707, (2019).
29 Grinberg, I. *et al.* Perovskite oxides for visible-light-absorbing ferroelectric and photovoltaic materials. *Nature* **503**, 509-512, (2013).
30 Yang, S. Y. *et al.* Above-bandgap voltages from ferroelectric photovoltaic devices. *Nat. Nanotechnol.* **5**, 143-147, (2010).
31 Quattropani, A. *et al.* Tuning photovoltaic response in Bi_2FeCrO_6 films by ferroelectric poling. *Nanoscale* **10**, 13761-13766, (2018).
32 Parameswaran, R. *et al.* Photoelectrochemical modulation of neuronal activity with free-standing coaxial silicon nanowires. *Nat. Nanotechnol.* **13**, 260-266, (2018).
33 Ghezzi, D. *et al.* A hybrid bioorganic interface for neuronal photoactivation. *Nat. Commun.* **2**, 166, (2011).
34 Chen, S. *et al.* Near-infrared deep brain stimulation via upconversion nanoparticle–mediated optogenetics. *Science* **359**, 679-684, (2018).
35 Lin, X. *et al.* Multiplexed optogenetic stimulation of neurons with spectrum-selective upconversion nanoparticles. *Adv. Healthcare Mater.* **6**, 1700446, (2017).
36 Miyazaki, T. *et al.* RETRACTED: large timescale interrogation of neuronal function by fiberless optogenetics using lanthanide micro-particles. *Cell Rep.* **26**, 1033-1043.e1035, (2019).
37 Hososhima, S. *et al.* Near-infrared (NIR) up-conversion optogenetics. *Sci. Rep.* **5**, 16533, (2015).
38 Wu, X. *et al.* Dye-sensitized core/active shell upconversion nanoparticles for optogenetics and bioimaging applications. *ACS Nano* **10**, 1060-1066, (2016).
39 Wu, X. *et al.* Tether-free photothermal deep-brain stimulation in freely behaving mice via wide-field illumination in the near-infrared-II window. *Nat. Biomed. Eng* **6**, 754-770, (2022).
40 Lyu, Y., Xie, C., Chechetka, S. A., Miyako, E. & Pu, K. Semiconducting polymer nanobioconjugates for targeted photothermal activation of neurons. *J. Am. Chem. Soc.* **138**, 9049-9052, (2016).
41 Nakatsuji, H. *et al.* Thermosensitive ion channel activation in single neuronal cells by using surface-engineered plasmonic nanoparticles. *Angew. Chem. Int. Ed.* **54**, 11725-11729, (2015).
42 Carvalho-de-Souza, João L. *et al.* Photosensitivity of neurons enabled by cell-targeted gold nanoparticles. *Neuron* **86**, 207-217, (2015).
43 Jiang, Y. *et al.* Heterogeneous silicon mesostructures for lipid-supported bioelectric interfaces. *Nat. Mater.* **15**, 1023-1030, (2016).
44 Jiang, Y. *et al.* Neural stimulation in vitro and in vivo by photoacoustic nanotransducers. *Matter* **4**, 654-674, (2021).
45 Zheng, N. *et al.* Photoacoustic carbon nanotubes embedded silk scaffolds for neural stimulation and regeneration. *ACS Nano* **16**, 2292-2305, (2022).
